# Cost-Effectiveness Analysis of a Bivalent Vaccine for Hand, Foot, and Mouth Disease: A Simulation-Based Study in Beijing, China

**DOI:** 10.3390/vaccines14010091

**Published:** 2026-01-17

**Authors:** Mengyao Li, Ying Shen, Yonghong Liu, Hui Yao, Zhuowei Luo, Da Huo, Xiang Xu, Wenhui Zhu, Shuaibing Dong, Lei Jia, Renqing Li, Bingyi Yang, Xiaoli Wang

**Affiliations:** 1 Beijing Key Laboratory of Surveillance, Early Warning and Pathogen Research on Emerging Infectious Diseases, Beijing Center for Disease Prevention and Control, Beijing 100013, China; limyao627@163.com (M.L.);; 2 School of Public Health, Capital Medical University, Beijing 100069, China; 3 School of Public Health, Peking University, Beijing 100191, China; 4 China Center for Health Development Studies, Peking University, Beijing 100084, China; 5 WHO Collaborating Centre for Infectious Disease Epidemiology and Control, School of Public Health, Li Ka Shing Faculty of Medicine, The University of Hong Kong, Hong Kong Special Administrative Region, China; 6 Public Health Emergency Management Innovation Center, Beijing 100013, China

**Keywords:** bivalent EV-A71/CV-A16 vaccine, vaccination strategy, cost-effectiveness analysis

## Abstract

Background: Hand, foot, and mouth disease (HFMD) remains a major public-health concern in China. While the monovalent EV-A71 vaccine has effectively reduced EV-A71–associated cases, it offers no protection against CV-A16. The introduction of a bivalent EV-A71/CV-A16 vaccine may offer broader protection, but its economic viability under different immunization strategies remains uncertain. Methods: We developed a dynamic transmission model integrated with cost-effectiveness analysis to assess the epidemiological and economic impact of a hypothetical bivalent EV-A71/CV-A16 vaccine in China. Based on the immunization program policy, seven vaccination strategies, vaccine effectiveness (VE) levels ranging from 50–95% against EV-A71/CV-A16, and coverage levels from 0–95% were evaluated. The threshold vaccine price (TVP) was derived based on incremental cost-effectiveness ratio (ICER) calculations. Cost-effectiveness was assessed using willingness-to-pay (WTP) thresholds defined as 1–3 times the gross domestic product (GDP) per capita. Results: The mean cost of two doses of the monovalent EV-A71 vaccine was USD133.0 (95% CI: 126.9–139.1). Strategy 2, which targeted individuals unvaccinated with the monovalent EV-A71 vaccine, demonstrated the most favorable cost-effectiveness. At 45% coverage and 85% vaccine effectiveness, the estimated threshold price per dose was USD 107.7 (95% CI: 103.4–112.0), with threshold vaccine prices increasing as coverage declined. When vaccination coverage exceeded 80%, the threshold vaccine price decreased substantially, falling below USD 45.9 (95% CI: 43.5–48.3) per dose. Conclusions: Large-scale inclusion in the national immunization program may not be economically justified at current cost levels. Targeted voluntary vaccination of unvaccinated, susceptible populations represents a more cost-effective and practical strategy during the early stage of vaccine introduction.

## 1. Introduction

Hand, foot, and mouth disease (HFMD) is a common pediatric illness primarily affecting children under five years of age, predominantly caused by enteroviruses EV-A71 and CV-A16 [1,2]. From 2008 to 2024, a total of approximately 24.68 million HFMD cases were reported in mainland China [3]. Although most HFMD cases are mild and self-limiting, a proportion can progress to severe complications, including encephalitis, myocarditis, and even death, highlighting the persistent threat HFMD poses to child health during periods of intense transmission [4,5]. In the absence of specific antiviral therapies, vaccination remains the most effective strategy for preventing HFMD and reducing its public health burden.

Since August 2016, three monovalent, inactivated EV-A71 vaccines (based on the C4 genotype) have been licensed in China. These vaccines are available on the domestic market for voluntary, self-paid immunization of children aged 6 months to 5 years [6,7]. A two-dose schedule, with a 28-day interval between doses, is recommended to induce effective immune immunity against EV-A71 infections [8]. Wang et al reported a vaccine effectiveness (VE) of 100% (95% CI: 68.1–100%) against severe and 83.7% (95% CI: 54.9–94.1%) against mild EV-A71 infections following a two-dose administration [8]. Furthermore, Xiao et al observed a 41.4% reduction in the incidence of EV-A71 infections following vaccine introduction [9]. A population-based study conducted in Pudong New Area of Shanghai further showed that, following the introduction of the inactivated EV-A71 vaccine, the average HFMD incidence rate declined markedly from 232.38 per 100,000 during 2012–2016 to 84.56 per 100,000 during 2017–2022 [10]. However, overall HFMD case counts remained high, largely due to the rising prevalence of other enteroviruses, particularly Coxsackievirus A6 (CV-A6) and A10 (CV-A10), which have become predominant serotypes [11]. In response, there is growing interest in developing bivalent or multivalent vaccines targeting multiple HFMD-associated enteroviruses. AIM Vaccine Co., Ltd. obtained the Clinical Trial Authorization (CTA) from the Center for Drug Evaluation (CDE) of the National Medical Products Administration (NMPA) for EV-A71-CV-A16 bivalent vaccine on October 2022 [12]. An EV-A71/CV-A16 bivalent inactivated vaccine developed by Sinovac completed a Phase I/II clinical trial. The Phase II clinical trial results showed that, 30 days post-vaccination, seroconversion rates against both EV-A71 and CV-A16 reached 100% in previously seronegative infants and young children across all low-, medium-, and high-dose groups of the bivalent vaccine [13]. The National Medical Products Administration (NMPA) granted approval for the clinical trial application of the quadrivalent inactivated enterovirus vaccine (Vero cell), developed by Sinovac in December 2024 [14].

Given that the bivalent vaccine is licensed and introduced, it will be critical to evaluate cost-effective vaccination strategies and pricing schemes. Although most CV-A16 infections manifest as mild cases [15], CV-A16 accounts for a relatively high proportion of HFMD cases. This imposes special considerations on the economic benefit assessment of the bivalent vaccine: compared with the EV-A71 vaccine, the benefits lie more in reducing the indirect burden from mild cases than in directly averting severe illness or death risks. Therefore, further research is needed to determine whether the introduction of the bivalent vaccine would be cost-effective and, if so, at what price point. However, there is currently a lack of research on the cost-effectiveness evaluation of the bivalent vaccine [16,17]. In response to this, we developed a comprehensive modeling framework that integrates real-world cost data with a dynamic transmission model to simulate multiple vaccination strategies and to estimate the corresponding threshold vaccine prices based on incremental cost-effectiveness analysis.

Furthermore, given the uncertainty surrounding the real-world effectiveness of the bivalent vaccine, and the fact that vaccine effectiveness in practice may be influenced by multiple factors, including vaccine coverage rates, baseline population immunity levels, circulating viral serotypes, and the age structure of the vaccinated population [18]. Therefore, a comprehensive model that incorporates varying assumptions regarding vaccine efficacy, cost, coverage, and immunization strategies is imperative to evaluate the cost-effectiveness of the bivalent vaccine under a range of plausible scenarios and to inform subsequent implementation policies and pricing.

In this study, we developed an age-structured dynamic transmission model to evaluate the cost-effectiveness of introducing a bivalent EV-A71/CV-A16 inactivated vaccine under diverse scenarios of efficacy, price, and coverage. By simulating the transmission dynamics of HFMD and incorporating real-world demographic and disease burden data, we aimed to identify optimal vaccination strategies and cost-effective pricing thresholds. The findings are intended to support evidence-based decision-making for the potential introduction of bivalent HFMD vaccines.

## 2. Methods

### 2.1. Data Sources

Daily data for HFMD cases from January 2011 to December 2024 were extracted from China’s National Notifiable Infectious Diseases Reporting Information System (NNIDRIS). Virological surveillance data, including serotype distribution, were obtained from the Beijing Center for Disease Prevention and Control. EV-A71 vaccination data, including coverage rates, were obtained from Beijing Management System of Information for the Immunization Program (BMSIIP). Immunogenicity was assumed to be conferred one month following the administration of the second vaccine dose. Demographic data, including population size and age structure, were collected from the Beijing Statistical Yearbook.

### 2.2. Cost

We conducted a structured questionnaire survey across six districts in Beijing, including two urban, two suburban, and two outer suburban districts to obtain the vaccination costs associated with EV-A71 vaccination. The survey targeted two groups: vaccination service providers and parents of vaccinated children. The survey aimed to quantify the full economic resources consumed throughout the vaccination process. The total cost comprised four types: (1) direct cost: the cost of the vaccine and administration, the cost of delivery, meals, accommodation, and other costs; (2) indirect cost: lost wages owing to vaccination; (3) cost of adverse events: medical costs, transportation, meals and lodging, and nursing fees owing to adverse reactions to the vaccine. Interviews were conducted at 16 community health care centers to determine their cost of vaccination, including the cost of injection, cold chain management, vaccine delivery, information system maintenance, program administration, health education, and adverse event management. An annual discount rate of 3% was applied to all costs and health outcomes.

The disease burden associated with HFMD was quantified based on estimates from published literature [16,19]. The health-associated outcomes of HFMD infection were measured in quality-adjusted life years (QALY) lost, which was obtained using the EuroQol five-dimension three-level questionnaire (EQ-5D-3L), EuroQol visual analogue scale (EQ-VAS) (Table S1), and the published literature [16,20]. A summary of all parameter values, including their sources, is provided in Table 1.

### 2.3. Transmission Models

To estimate the cost-effectiveness of different bivalent vaccination strategies, we employed a two-step modeling approach. First, a multiplier model was applied to account for under-reporting and derive the actual infection numbers from surveillance data. Key parameters for this model, including the baseline infection rate and the distribution of illness severity, were calibrated based on estimates from Shen et al. [19] (Table 1). The time series of daily infections was divided into three age groups (0–2 years, 3–5 years, ≥6 years).

Subsequently, an age-structured, compartmental transmission model Susceptible–Exposed–Infectious–Removed–Vaccinated (SEIRV) was constructed to estimate the impact of different vaccination strategies. The compartments are defined as follows:

S (Susceptible): Individuals at risk of infection.

E (Exposed): Individuals infected but not yet infectious.

I (Infectious): Individuals actively infectious and capable of transmitting the virus.

R (Removed): Individuals who have recovered and acquired immunity.

V (Vaccinated): Individuals who have been vaccinated.

The transitions between these compartments for each age group i are governed by the following set of differential equations:

For each *i* ∈ {1,2,3}:

$$\begin{array}{cc} \; & \frac{dS_{i}}{dt}=B_{i}-\lambda_{i}\left(t\right)S_{i}-v_{i}\left(t\right)+\rho S_{i-1}-\rho S_{i}\\ \; & \frac{dV_{i}}{dt}=v_{i}\left(t\right)-\lambda_{i}\left(t\right)V_{i}\left(1-VE\right)+\rho V_{i-1}-\rho V_{i}\\ \; & \frac{dE_{i}}{dt}=\lambda_{i}\left(t\right)S_{i}+\lambda_{i}\left(t\right)V_{i}\left(1-VE\right)-\sigma E_{i}+\rho E_{i-1}-\rho E_{i}\\ \; & \frac{dI_{i}}{dt}=\sigma E_{i}-\gamma I_{i}+\rho I_{i-1}-\rho I_{i}\\ \; & \frac{dR_{i}}{dt}=\gamma I_{i}+\rho R_{i-1}-\rho R_{i}\\ \; & \lambda_{i}\left(t\right)=\beta_{i}\left(1+\alpha \cos\left(\frac{2\pi t}{365}+\theta \right)\right)\sum_{j=1}^{3}C_{ij}\frac{I_{j}}{N_{j}}\\ \; & N_{i}=S_{i}+E_{i}+I_{i}+R_{i}+V_{i}\\ \; & C=\left(\begin{array}{ccc} c_{11} & c_{12} & c_{13}\\ c_{12} & c_{22} & c_{23}\\ c_{13} & c_{23} & c_{33} \end{array}\right) \end{array}$$

### 2.4. Vaccination Simulation Scenarios

We conducted simulations from three key dimensions: vaccination strategy (from strategy 1–7), vaccination coverage (from 0–95%), and VE (from 50–95%). Five vaccination strategies were defined as:

Strategy 1 (Universal Voluntary): All eligible individuals receive the bivalent vaccine at a specified voluntary coverage rate, irrespective of their prior monovalent EV-A71 vaccination status.

Strategy 2 (Monovalent-Exempt): Only individuals who have not previously received the monovalent EV-A71 vaccine are eligible for the bivalent vaccine, which is administered at a voluntary coverage rate.

Strategy 3 (Routine Birth-Cohort): Infants below one year of age are routinely vaccinated with 100% coverage as part of the standard immunization schedule.

Strategy 4 (Birth-Cohort + 1–2 Catch-Up): Routine vaccination of newborns is supplemented by a one-time catch-up campaign targeting children aged 1–2 years.

Strategy 5 (Birth-Cohort + 1–5 Catch-Up): Routine vaccination of newborns is combined with a one-time catch-up campaign for all children aged 1–5 years.

Strategy 6 (Birth-Cohort + 3–5 Catch-Up): Routine vaccination of newborns is supplemented by a one-time catch-up campaign targeting children aged 3–5 years.

Strategy 7 (Birth-Cohort + Voluntary): Routine vaccination of newborns is combined with voluntary vaccination among children aged 1–5 years, administered at the same voluntary coverage rates as previously assumed for voluntary vaccination strategies.

### 2.5. Cost Effectiveness Analysis

Health benefits were expressed as QALYs averted relative to a monovalent EV-A71 baseline. Incremental cost-effectiveness ratios (ICERs) were calculated to compare vaccination strategies.

$$C=C^{burden}+C^{vaccine}\;ICER=\frac{C_{bi}-C_{mono}}{{QALYs}_{bi}-{QALYs}_{mono}}$$

C is the total cost under each strategy and is defined as the sum of disease burden costs $C^{burden}$ and vaccination program costs $C^{vaccine}$. $C_{bi}$ and $C_{mono}$ denote the total costs under the bivalent and monovalent vaccination strategies, respectively, and ${QALYs}_{bi}$ and ${QALYs}_{mono}$ denote their corresponding health outcomes. According to WHO standard: ICER < GDP per capita means highly cost-effective, GDP per capita < ICER < 3 × GDP per capita means cost-effective and ICER > 3 × GDP per capita means not cost-effective. When the ICER equals the willingness-to-pay (WTP) threshold, the threshold vaccine price (TVP) of the bivalent vaccine per dose is given by,

$$TVP=\frac{{WTP\times (QALYs}_{bi}-{QALYs}_{mono})+C_{mono}-C_{bi}^{medical}}{N_{dose}}-C_{bi}^{other}$$

$C_{bi}^{medical}$ represents the retained medical burden cost under the bivalent strategy, $C_{bi}^{other}$ denotes the per-dose non-price vaccination cost, and $N_{dose}$ is the total number of bivalent vaccine doses administered. All statistical analyses were carried out by using R 4.3.1 software.

## 3. Results

**Figure 1:**
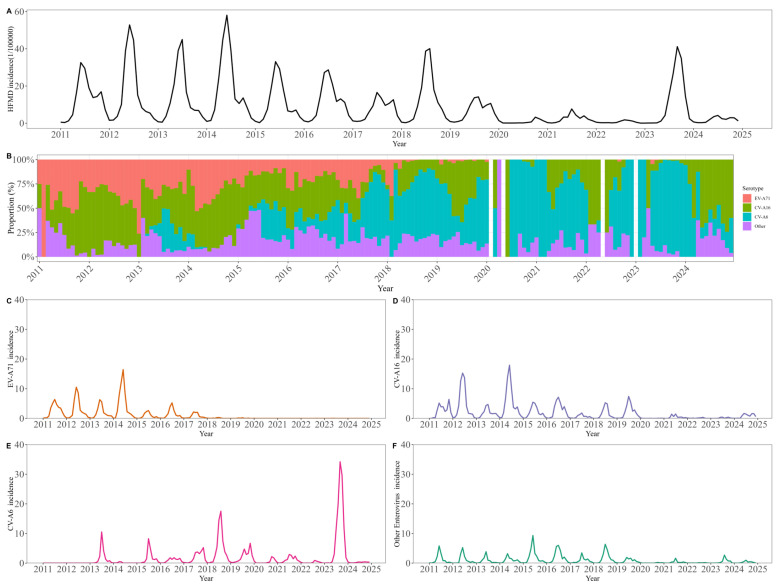
Annual incidence and serotype-specific positivity rates of hand, foot, and mouth disease in Beijing, 2011–2024. (**A**) The monthly incidence of HFMD from 2011 to 2024. (**B**) Serotype composition of HFMD from 2011 to 2024. (**C**–**F**) The monthly incidence of EV-A71, CV-A16, CV-A6 and other enteroviruses from 2011 to 2024.

Between 2011 and 2024, a total of 325,383 hand, foot, and mouth disease (HFMD) cases were reported in Beijing, corresponding to an overall incidence of 106.4 per 100,000 population annually (Figure 1). Following the introduction of the EV-A71 vaccine in 2016, the overall HFMD incidence exhibited a general declining trend, interrupted only by a transient increase in 2018. This epidemiological shift was accompanied by a marked reduction in EV-A71 positivity among cases and a substantial increase in CV-A16 and CV-A6 positive rates. A total of 1095 severe cases and 14 deaths were reported in 14 years. The number of severe HFMD cases and deaths has declined since 2012 (Figure S1).

The mean total cost of the two-dose EV-A71 vaccine was USD 133.0 (95% CI: USD 126.9–139.1) (Table 2). The price of the two-dose vaccine was USD 54.2, which accounted for 40.8% of the cost. The mean cost of the two-dose vaccine paid by the family was USD120.4 (95% CI: USD 114.8–126.0), which mainly comprised the direct medical cost (USD 71.6, 59.5%) and indirect cost (USD 51.8, 43.0%). The mean cost paid by community health care centers was USD12.6 (95% CI: USD 12.0–13.2).

**Figure 2:**
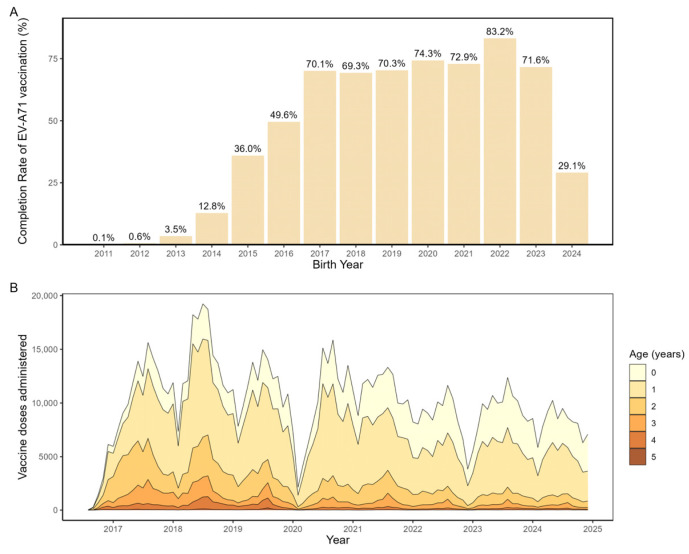
EV-A71 Vaccination status in Beijing during 2016–2024. (**A**) Vaccination Coverage by birth cohort. The drop in the 2024 birth cohort is primarily driven by right censoring. Detailed calculation methods are available in Text S1; (**B**) Age-stratified monthly distribution of the second dose of EV-A71 vaccine.

From 2011 to 2024, a total of 1,026,643 children in Beijing completed the full EV-A71 vaccination series, corresponding to an overall coverage rate of 43.7%. Cohort analysis (Figure 2A) showed higher vaccination rates among birth cohorts from 2016 to 2023 (range: 69.3–82.3%) compared with cohorts born between 2011 and 2015 (all below 50%). Analysis of monthly administrations of the second dose of EV-A71 vaccine by age group (Figure 2B) indicated that vaccination was predominantly concentrated within the first year of life, peaking between 6 and 12 months of age, followed by a sharp decline after the first birthday and only sporadic catch-up vaccination in older children.

**Figure 3:**
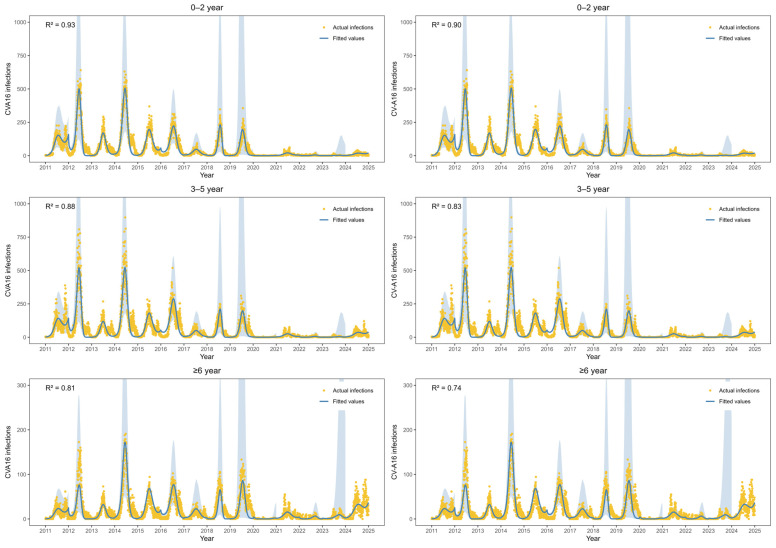
Observed versus predicted EV-A71 and CV-A16 infections over time, stratified by age group. (the blue shading indicates the 95% confidence intervals of the fitted values).

Figure 3 shows the fitted values of EV-A71 infections and CV-A16 infections from 2011 to 2024. The estimated parameters are in Table S2. For EV-A71 infections, the R^2^ values in three age groups were 0.93, 0.88 and 0.81, respectively. For CV-A16 infections, the R^2^ values in three age groups were 0.90, 0.83 and 0.74, respectively. CV-A16 infections exhibited a distinct biennial pattern during 2011–2019, with higher incidence observed in even-numbered years. After adjustment using the multiplicative model to account for underreporting and non-consultation, the estimated mean annual number of CV-A16 infections during this period was 608,905 cases. During 2020–2022, CV-A16 infections remained at a relatively low level. In contrast, a marked increase in CV-A16 infections was observed among individuals aged ≥6 years during 2023–2024, with an average of 4997 infections per year, accounting for 44.5% of all CV-A16 infections over the two-year period.

**Figure 4:**
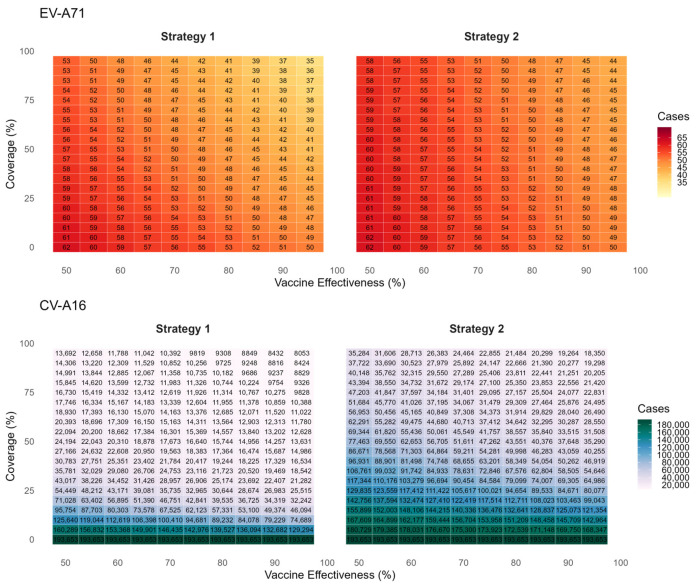
Simulated cumulative EV-A71 and CV-A16 infections from 2025 to 2029 under Strategies 1 and 2.

Figure 4 presents the predicted cumulative infections of EV-A71 and CV-A16 over the next five years under voluntary vaccination strategies (Strategy 1 and Strategy 2). The simulation results indicate that EV-A71 infection will remain at a low incidence level, and the coverage and protection effectiveness of the bivalent vaccine have only a limited impact on EV-A71 transmission. Compared with no vaccination, achieving 95% coverage and 95% effectiveness would reduce EV-A71 infections by 27 cases. In contrast, the average annual number of CV-A16 infections is projected to be 38,731. Under an assumption of 85% vaccine effectiveness and 45% coverage (aligning with current Beijing levels), Strategy 1 is estimated to result in 16,474 CV-A16 infections—a 91.5% reduction versus no vaccination. Strategy 2, which excludes previously monovalent-vaccinated children, shows a less pronounced effect, with 46,283 infections and a 76.1% reduction.

**Figure 5:**
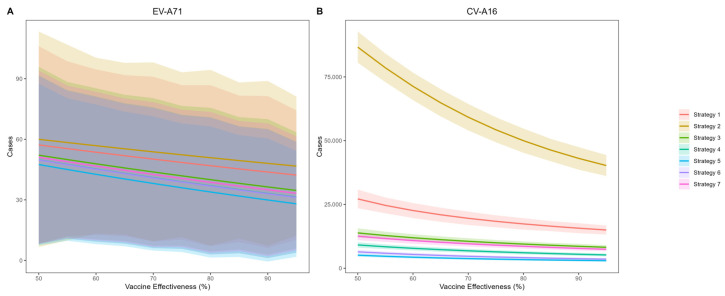
Projected Cumulative EV-A71 (**A**) and CV-A16 (**B**) Infections under Alternative Vaccination Strategies 1–7.

Assuming a voluntary uptake of 45% in Strategies 1 and 2 based on Beijing’s historical coverage, we compared EV-A71 and CV-A16 infections across all five vaccination strategies at varying vaccine effectiveness levels (Figure 5). The results demonstrate that CV-A16 incidence under Strategy 2 was substantially higher than under the other strategies. Assuming 85% protection effectiveness against CV-A16, the overall effectiveness in reducing infections for Strategies 1 through 7 was estimated at 91.5% (95% CI: 89.0–94.0%), 76.1% (95% CI: 72.1–80.1%), 95.3% (95% CI: 94.1–96.3%), 97.0% (95% CI: 96.3–97.7%), 98.3% (95% CI: 97.9–98.7%), 98.0% (95% CI: 97.7–98.2%) and 95.8% (95% CI: 95.4–96.2%) respectively, compared with the no-vaccination scenario.

**Figure 6:**
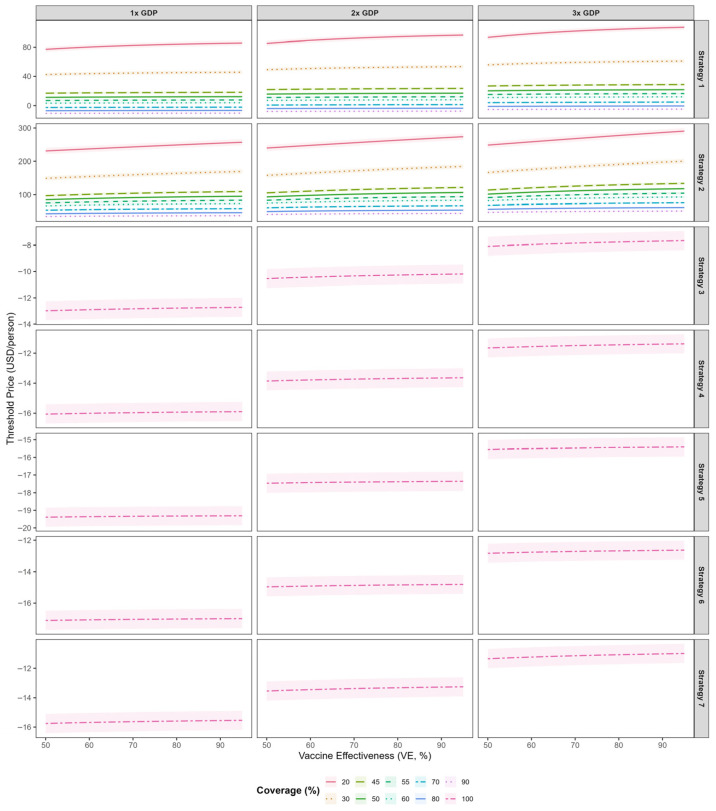
Threshold Vaccine Price for Bivalent EV-A71/CV-A16 Vaccine under Different Strategies and Willingness-to-Pay Levels.

Figure 6 presents the threshold vaccine price (TVP) per dose for the bivalent EV-A71/CV-A16 vaccine across five immunization strategies at varying willingness-to-pay (WTP) thresholds. Across all strategies, threshold vaccine prices increased with higher vaccine effectiveness. For strategies that incorporated the bivalent vaccine into the national immunization program, the TVPs were negative when evaluated against WTP thresholds of 1–3 times GDP per capita, indicating a lack of cost-effectiveness under these scenarios. Among the NIP-based strategies, Strategy 3 yielded relatively higher TVP values; at a vaccine effectiveness of 0.85, the TVP was −12.8 USD (95% CI: −13.5--12.0). Among voluntary vaccination strategies, Strategy 2 consistently supported higher threshold prices than Strategy 1. At 45% coverage and 85% vaccine effectiveness, the per-dose TVPs under WTP thresholds of 1×, 2×, and 3× GDP per capita were estimated at 107.7 USD (95% CI: 103.4–112.0), 119.5 USD (95% CI: 115.3–123.8), and 131.3 USD (95% CI: 127.1–135.6), respectively. At 80% coverage and 85% vaccine effectiveness, the corresponding per-dose TVPs decreased to 45.9 USD (95% CI: 43.5–48.3), 53.5 USD (95% CI: 51.1–55.9), and 61.1 USD (95% CI: 58.7–63.5), respectively. Compared with higher coverage levels, threshold vaccine prices were higher under lower coverage conditions. Threshold vaccine costs under the various scenarios are presented in Table S3.

## 4. Discussions

In this study, we estimated the maximum threshold vaccine cost (TVC) for a hypothetical bivalent EV-A71/CV-A16 vaccine relative to the existing monovalent EV-A71 vaccine, across five deployment strategies, a range of vaccine effectiveness (5–95%), and coverage levels (50–95%). By integrating dynamic transmission modeling, health utility losses (QALYs), direct and indirect costs, and willingness-to-pay benchmarks, we assessed cost-effectiveness of each strategy in HFMD burden. The results showed that incorporating the bivalent vaccine into the national immunization program could achieve the largest reduction in CV-A16 infections (97.0%), but it was not cost-effective. Strategy 2 (limited to individuals not previously vaccinated with the monovalent EV-A71 vaccine) proved to be the most cost-effective. After excluding delivery and programmatic costs, the pure vaccine component threshold price was estimated at USD 107.7 (95% CI:103.4–112.0) at 45% coverage. Threshold vaccine prices increased as coverage declined, indicating that each vaccinated individual provided greater incremental value under low-coverage conditions and underscoring that even modest increases in uptake can yield substantial socioeconomic benefits.

Our analysis found that EV-A71 vaccine coverage in Beijing from 2011 to 2024 was 43.7%, which was consistent with the 42.78% reported by Zhang et al. [23] and was higher than the national vaccine coverage rate of 24.96% [23]. Several factors may explain why vaccine uptake in Beijing is markedly higher than the national average. First, Urban districts benefit from denser clinic networks, reducing missed opportunities and dropouts. Second, targeted public health campaigns and strong community engagement in the capital may have driven greater parental awareness of and acceptance of the EV-A71 vaccine [24]. Third, Zhang et al have found that equity of non-NIP vaccine uptake in China was contributed by monthly family income per capita and education level [25]. Given that Beijing ranks highly in both income and educational attainment, these socioeconomic advantages likely contributed to the higher EV-A71 vaccine coverage.

For CV-A16, the age-specific infection patterns of CV-A16 further reflect important shifts in the epidemiology of HFMD in Beijing. In recent years, the age distribution of HFMD cases has shown a clear tendency toward older age groups, with individuals aged ≥6 years accounting for an increasing proportion of reported cases [26]. In 2023, the incidence of HFMD among individuals aged ≥6 years reached 73.7 per 100,000, substantially exceeding the levels observed in previous years (2.7–28.7 per 100,000). Meanwhile, CV-A16 re-emerged as the predominant circulating serotype in 2024, further amplifying the disease burden attributable to CV-A16 in older age groups. These findings suggest a structural shift toward older-age susceptibility in HFMD transmission; however, the underlying drivers of this age shift warrant further investigation integrating behavioral, immunological, and virological evidence.

In our simulation, with a vaccine effectiveness of 85% and coverage of 45%, the bivalent vaccine reduced CV-A16 infections by 76.1%. This substantial reduction achieved under moderate coverage suggests that even limited vaccine uptake can effectively interrupt transmission chains. Our findings are consistent with those of Liu et al. [27], who reported a 70.6% reduction in cases when the bivalent vaccine had a VE of 80% and a coverage rate of 50%. This phenomenon aligns with the theoretical framework proposed by Skene et al. [28], who described similar diminishing marginal returns in influenza vaccination programs. They demonstrated that the marginal benefit of vaccinating additional individuals increases until herd immunity is approached, after which it drops sharply.

Our findings showed that, compared with the monovalent EV-A71 vaccine, the bivalent EV-A71/CV-A16 vaccine was more cost-effective under certain scenarios, which is consistent with the results reported by Liu et al. [27]. However, in terms of strategy selection, incorporating the bivalent vaccine into the national immunization program, although achieving the greatest reduction in cases from an epidemiological perspective, was not cost-effective from an economic perspective even if the vaccine were provided free of charge. This may be attributable to the substantial additional vaccine doses and extensive programmatic and implementation resources required, which led to markedly increased total costs. Among the voluntary vaccination strategies, Strategy 2 —targeting only those not previously vaccinated with the monovalent EV-A71 vaccine—demonstrated the most favorable cost-effectiveness profile. This suggests that focusing on susceptible and unvaccinated populations can achieve the greatest health benefit per unit cost. In terms of pricing, assuming that the bivalent vaccine has similar vaccine effectiveness and coverage to the existing EV-A71 vaccine, our study estimated a threshold price per dose of 107.7 (95% CI: 103.4–112.0). This value is substantially higher than that reported by Liu et al. [27]. The discrepancy may be explained by differences in model structure and parameterization. Specifically, our analysis employed a dynamic transmission model that incorporated the true population-level infection burden using a multiplicative framework, accounting for unreported and unconsulted infections that were not captured in surveillance-based studies [29]. By explicitly including these hidden cases and their associated health and economic burdens, our model yielded a higher overall disease burden baseline, thereby increasing the estimated threshold vaccine price required for cost-effectiveness. Under Strategy 2, lower vaccination coverage was associated with higher threshold vaccine prices (TVPs). This pattern may be explained by the diminishing marginal returns of vaccination. At lower coverage levels, newly vaccinated individuals are predominantly susceptible and unvaccinated, so each additional dose yields relatively large incremental health benefits, thereby supporting a higher acceptable vaccine price per dose. As coverage increases, a growing proportion of vaccinated individuals have lower baseline infection risk or prior immunity, resulting in reduced marginal health gains per additional dose and, consequently, lower threshold vaccine prices.

Although indirect herd immunity effects were not explicitly incorporated into the current model, their potential implications for cost-effectiveness outcomes warrant further discussion. Previous vaccine cost-effectiveness studies using dynamic transmission models have consistently shown that when vaccination coverage exceeds a critical threshold, transmission cannot be sustained because the number of susceptible individuals becomes insufficient, thereby reducing infection risk even among unvaccinated individuals [30]. Incorporating indirect protection through herd immunity and reduced transmission can substantially increase population-level health gains and yield lower ICERs compared with analyses that omit these effects. Such herd immunity effects have been well documented for multiple vaccination programs, including influenza and pneumococcal. Therefore, if indirect protective effects were considered in the present study, the cost-effectiveness profile of the bivalent vaccine would be expected to improve further, potentially supporting a higher threshold vaccine price under the same willingness-to-pay criteria. This suggests that the current estimates may be conservative.

However, the generalizability of our findings should be interpreted with consideration of two contextual factors. First, this analysis assessed the bivalent EV-A71/CV-A16 vaccine relative to an existing monovalent EV-A71 vaccination baseline. In settings where an EV-A71 immunization program has not yet been implemented, the baseline burden of HFMD is likely to be higher, particularly with respect to severe disease and mortality associated with EV-A71, and the introduction of a bivalent vaccine may therefore generate greater incremental health benefits. Second, this study was conducted using Beijing-specific data from a large metropolitan area with relatively high healthcare utilization, medical expenditures, and willingness to pay. Consequently, the threshold vaccine prices estimated in this study may be higher than those applicable to regions with lower economic resources or different healthcare cost structures. Future studies should conduct similar analyses in settings with different socioeconomic levels to assess the generalizability of these findings.

Several limitations should be noted. First, the estimates of HFMD disease burden and QALY losses were derived from population-level assessments that pooled all HFMD cases, regardless of etiological agent. Consequently, potential heterogeneity in disease severity and health-related quality of life across different enterovirus serotypes. Second, the baseline cost estimates were derived from published data with uncertainty ranges; while sensitivity analyses using low and high values were included, real-world costs may differ across healthcare settings. Third, indirect benefits such as herd immunity and reduced transmission dynamics were not explicitly incorporated. Inclusion of these indirect effects would be expected to increase overall health gains, reduce ICERs, and consequently allow for a higher threshold vaccine cost under the same willingness-to-pay criteria, suggesting that our estimates may be conservative. Fourth, the analysis assumed constant vaccine effectiveness over time and did not account for waning immunity, which may reduce long-term protection and alter cost-effectiveness outcomes. In addition, potential serotype competition and replacement effects from non-vaccine enteroviruses, such as CV-A6 and CV-A10, were not explicitly modeled. Shifts in serotype dominance could influence future disease burden and may affect the long-term economic value of bivalent vaccination strategies. These factors warrant further investigation using long-term surveillance data and multi-serotype dynamic transmission models.

## 5. Conclusions

This study systematically evaluated the potential epidemiological and economic impacts of introducing a bivalent EV-A71/CV-A16 vaccine in Beijing by integrating dynamic transmission modeling with cost-effectiveness analysis. The results showed that even at moderate coverage levels, the bivalent vaccine could substantially reduce the burden of HFMD. Among the evaluated immunization strategies, voluntary vaccination targeting individuals who had not previously received the monovalent EV-A71 vaccine proved to be the most cost-effective, suggesting that, during the early phase of vaccine introduction, precision immunization strategies focusing on susceptible populations should be prioritized.

## Figures and Tables

**Table 1:** Parameters used in the cost-effectiveness analysis.

Parameter	Definitions	Value
The Multiplier model		
Actual infection rate	Estimated number of actual infections represented by one laboratory-confirmed HFMD case	9.1 (4.6, 31.5) [19]
The proportion of un-consulted	Estimated fraction of infected individuals who do not seek medical consultation despite experiencing symptoms.	78.02% [19]
The proportion of misdiagnosed	Estimated fraction of individuals who seek medical care but are incorrectly diagnosed with diseases other than HFMD despite being truly infected.	9.6% [19]
The proportion of mild cases	Estimated fraction of HFMD infections presenting with mild or subclinical symptoms	11.0% [19]
The proportion of severe cases	Estimated fraction of HFMD infections that progress to severe disease	EV-A71: 1.2% CV-A16: 0.3%
The proportion of fatal cases	Estimated fraction of HFMD infections that result in death among all infected individuals	EV-A71: 0.18% CV-A16: 0
MSEIRV model		
$B_{i}$	the daily inflow of newborns	Population aged 0 year/365
$\lambda_{i}\left(t\right)$	the force of infection	-
$\beta_{i}$	Baseline transmission coefficient for age group *i*	Estimated
α	Amplitude of seasonal variation	Estimated
θ	D Parameter determining the timing of the seasonal peak transmission	Estimated
ρ	Ageing rate	1/(365 × 3)
$v_{i}\left(t\right)$	Per-capita vaccination hazard in age group i	-
σ	Progression rate from exposed to infectious	1/4 [21]
γ	Removal rate from infectious	1/7 [21]
VE	Vaccine Effectiveness	EV-A71 vaccine: 83.7% [8] BivalentVaccine: 50%, 55%, 60%, 65%, 70%, 75%, 80%, 95%
C	Symmetric contact-matrix	$\left(\begin{array}{ccc} 1 & 0.56 & 1.85\\ 0.56 & 1 & 1.85\\ 1.85 & 1.85 & 1 \end{array}\right)$ [22]
HFMD Disease Burden		
Unconsulted	Infected individuals who do not seek medical consultation despite experiencing symptoms	27.95 (8.56, 47.39) [19]
Diagnosed as other	Individuals who seek medical care but are incorrectly diagnosed with diseases other than HFMD despite being truly infected	290.39 (232.31, 348.47) [19]
Mild	-	400.48 (368.42, 432.57) [19]
Severe	-	2574.34 (1716.46, 3431.98) [19]
Fatal	-	4138.02 (3035.56, 5241.78) [22]
QALYs		
Severe		0.0194 [16]
Fatal		30.4 [16]
Mild and Unconsulted		0.0014

**Table 2:** Mean cost of a single dose of EV-A71 vaccine (USD).

Variable	Mean	SD	Median (IQR)	Range
Price of Vaccine	27.1	1.4	27.8 (24.8, 27.8)	(24.8, 29.5)
For parents/families of vaccinated subjects				
Direct Cost	34.3	11.6	33.0 (28.5, 34.5)	(28.5, 181.3)
Indirect Cost	25.9	47.6	14.2 (0.0, 35.5)	(0.0, 1064.8)
Cost for adverse events	0.0	1.0	0.0 (0.0, 0.0)	(0.0, 26.6)
Sub Total	60.2	48.9	49.2 (33.7, 68.5)	(28.5, 1096.3)
For community healthcare center				
Sub Total	6.3	5.0	5.2 (4.1, 6.4)	(1.5, 22.9)
Total	66.5	53.9	54.4 (37.8, 74.9)	(30.0, 1119.2)

The average exchange rate for RMB to USD was taken as 0.1394 in the year 2024. EV, enterovirus; SD, standard deviation; IQR, interquartile range. “Direct Cost” includes the vaccine price.

## Data Availability

The original contributions presented in this study are included in the article/Supplementary Material.

## Supplementary Materials

The following supporting information can be downloaded at: https://www.mdpi.com/article/10.3390/vaccines14010091/s1. Text S1: Method for Estimating EV-A71 Vaccination Coverage by Birth Cohort; Figure S1: Annual Trends and Laboratory Composition of Severe and Fatal HFMD Cases; Table S1: Loss of QALYs according to the EQ-5d-3L and EQ-VAS; Table S2: The estimated parameters of MSEIRV models from 2011 to 2024; Table S3: Threshold Vaccine Price for Bivalent EV-A71/CV-A16 Vaccine under Different Strategies and Willingness-to-Pay Levels.
