# Supplementary material for: Cost-Effectiveness Analysis of a Bivalent Vaccine for Hand, Foot, and Mouth Disease: A Simulation-Based Study in Beijing, China
Source: Vaccines (Basel). 2026 Jan 17;14(1):91. doi: 10.3390/vaccines14010091 (PMC12846606; doi:10.3390/vaccines14010091)
Supplement: Supplementary file 1 [file vaccines-14-00091-s001.zip › vaccines-4042783-supplementary.pdf]

Text S1

Vaccination coverage of EV-A71 was estimated based on a birth-cohort approach rather than using annual population denominators. Specifically, for each birth year cohort, we identified the number of children who completed the full EV-A71 vaccination schedule during the eligible age window (0-5 year). The cohort-based coverage was then calculated as the proportion of children within the same birth cohort who received the complete vaccination series. The vaccination coverage for birth cohort b is calculated as:

$$Coverage_b = \frac{C_b}{N_b}$$

$$C_b = \sum_{a=0}^{4} V_{b,a}$$

C_b denote the number of children born in year b who **c**ompleted the full vaccination schedule, and N_b denote the total number of births in the same birth cohort. $V_{b,a}$ represents the number of children born in year b who completed the full vaccination schedule at age a (with a measured in years, covering ages 0 to 4).

Table S1 Loss of QALYs according to the EQ-5d-3L and EQ-VAS

Burdon	No problem	Moderate problem	Severe problem
Mobility	83.0%	16.1%	0.9%
Self-care	84.9%	14.3%	0.9%
Usual activities	78.0%	20.8%	1.2%
Pain/discomfort	38.7%	57.8%	3.4%
Anxiety/depression	80.8%	18.6%	0.6%
Scores		93.6±10.3	
QALY		0.0014	

QALY, quality-adjusted life year; EQ-5D-3L, EuroQol five-dimension three-level questionnaire; EQ-VAS, EuroQol visual analogue scale.

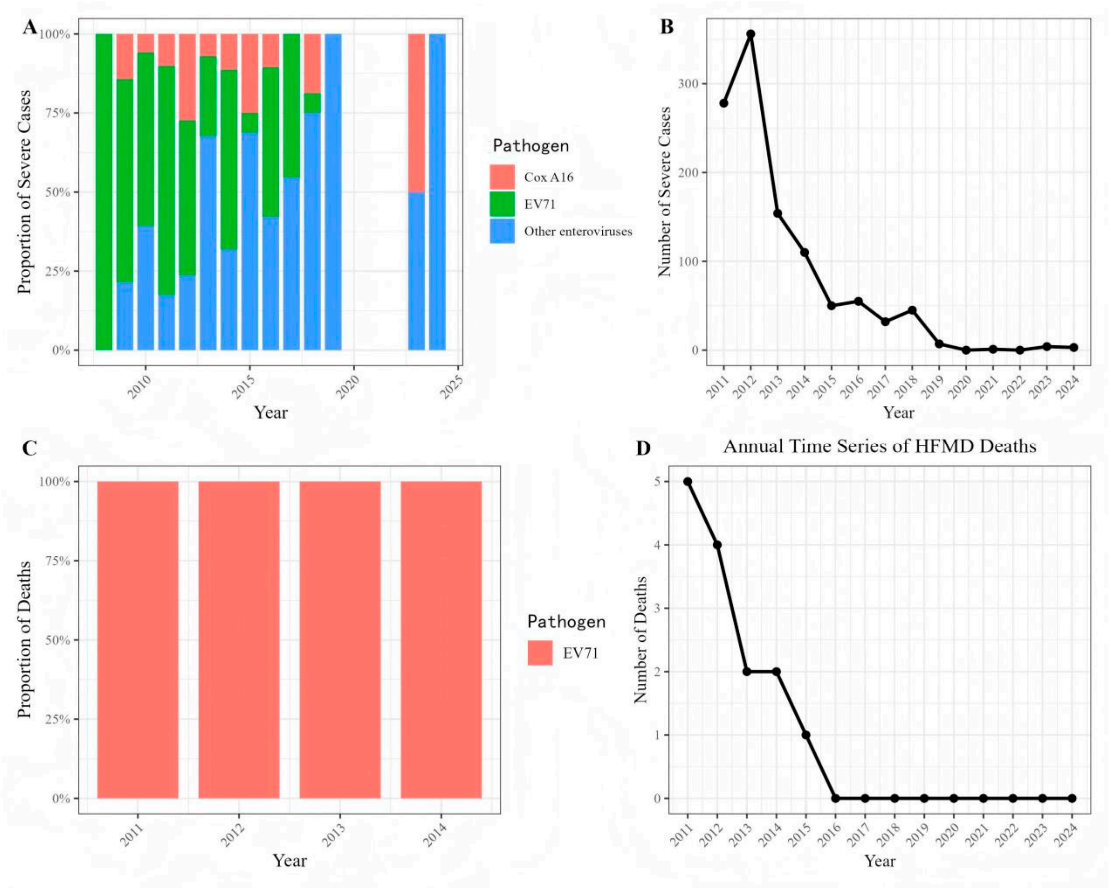

Figure S1. Annual Trends and Laboratory Composition of Severe and Fatal HFMD Cases A. Yearly Composition of Lab Results for Severe HFMD Cases; B. Annual Time Series of Severe HFMD Cases; C. Yearly Composition of Lab Results for HFMD Deaths; D. Annual Time Series of HFMD Deaths

Table S2 The estimated parameters of MSEIRV models from 2011 to2024

Year	β_1	β_2	β_3	α	θ
EV-A71					
2011	0.10730 (0.10677, 0.11329)	0.12047 (0.11050, 0.12148)	0.00020 (0.00004, 0.00022)	-0.20860 (-0.26132, -0.20265)	1.53989 (1.53330, 1.62484)
2012	0.07237 (0.05182, 0.08547)	0.08414 (0.07678, 0.09309)	0.00009 (0.00000, 0.00024)	-0.91651 (-1.12071, -0.70367)	1.17436 (1.11684, 1.25621)
2013	0.10723 (0.08109, 0.12420)	0.11211 (0.08893, 0.12706)	0.00024 (0.00011, 0.00037)	-0.53559 (-1.13897, -0.31760)	1.58032 (1.22912, 1.77210)
2014	0.10429 (0.10030, 0.10927)	0.15596 (0.15493, 0.15810)	0.00025 (0.00016, 0.00034)	-0.37179 (-0.38736, -0.32769)	2.27272 (2.18080, 2.36941)
2015	0.08899 (0.07848, 0.09468)	0.10153 (0.09187, 0.10602)	0.00018 (0.00002, 0.00033)	-0.55917 (-0.68070, -0.45635)	1.03633 (0.95864, 1.12704)
2016	0.10196 (0.10093, 0.10246)	0.14247 (0.13642, 0.14451)	0.00052 (0.00042, 0.00056)	-0.33332 (-0.37120, -0.31891)	1.65020 (1.53202, 1.69179)
2017	0.09570 (0.08685, 0.09850)	0.11196 (0.10441, 0.12549)	0.00026 (0.00013, 0.00045)	-0.51354 (-0.59435, -0.36852)	0.94313 (0.85218, 1.10342)
2018	0.09607 (0.09607, 0.09607)	0.08330 (0.08330, 0.08330)	0.00034 (0.00034, 0.00034)	-0.95891 (-0.95891, -0.95891)	0.89625 (0.89625, 0.89625)
2019	0.08138 (0.08104, 0.09159)	0.09300 (0.07540, 0.09359)	0.00031 (0.00031, 0.00043)	-1.14535 (-1.22463, -1.14269)	0.79416 (0.77286, 0.79487)
2020	0.02855 (0.02804, 0.02954)	0.02005 (0.01760, 0.02554)	0.00031 (0.00029, 0.00043)	2.31609 (1.80105, 2.43988)	0.42947 (0.35118, 0.55582)
2021	0.00348 (0.00019, 0.00603)	0.00094 (0.00016, 0.00392)	0.00033 (0.00001, 0.00071)	-1.15757 (-1.67928, -0.39250)	0.11186 (-0.25934, 0.45181)

2022	0.03741 (0.03182, 0.04748)	0.03593 (0.03394, 0.03737)	0.00019 (0.00002, 0.00023)	-3.32504 (-3.53537, -3.03032)	-0.91206 (-0.92102, -0.87256)
2023	0.04950 (0.03656, 0.06057)	0.05923 (0.05179, 0.07397)	0.00032 (0.00019, 0.00040)	-2.56378 (-2.85329, -1.93244)	0.93279 (0.89818, 1.05911)
2024	0.00360 (0.00061, 0.01094)	0.00157 (0.00037, 0.00369)	0.00047 (0.00007, 0.00073)	-1.09096 (-2.01775, -0.53382)	-0.07336 (-0.66917, 0.22692)
CV-A16					
2011	0.11614 (0.10223, 0.11759)	0.11902 (0.11771, 0.13163)	0.00015 (0.00003, 0.00016)	-0.16633 (-0.16940, -0.13678)	1.92480 (1.91857, 1.92545)
2012	0.07291 (0.06842, 0.07609)	0.08594 (0.07922, 0.09467)	0.00009 (0.00004, 0.00011)	-0.83467 (-0.98780, -0.73613)	1.21389 (1.18036, 1.29038)
2013	0.12736 (0.12741, 0.12741)	0.11969 (0.11973, 0.11973)	0.00021 (0.00021, 0.00021)	-0.33073 (-0.32999, -0.32999)	1.72482 (1.72636, 1.72636)
2014	0.10069 (0.10064, 0.10072)	0.13161 (0.12594, 0.13879)	0.00030 (0.00024, 0.00036)	-0.37695 (-0.42230, -0.31946)	1.73557 (1.65149, 1.84216)
2015	0.12024 (0.12029, 0.12029)	0.14656 (0.14662, 0.14662)	0.00037 (0.00037, 0.00037)	-0.23312 (-0.23218, -0.23218)	1.87103 (1.87148, 1.87148)
2016	0.09095 (0.08926, 0.09587)	0.14483 (0.14404, 0.14713)	0.00028 (0.00026, 0.00035)	-0.27640 (-0.30267, -0.19969)	0.93653 (0.93122, 0.95204)
2017	0.10204 (0.10199, 0.10207)	0.13941 (0.13374, 0.14660)	0.00046 (0.00040, 0.00052)	-0.21699 (-0.26234, -0.15950)	1.28705 (1.20296, 1.39363)
2018	0.08577 (0.08234, 0.09713)	0.10050 (0.09966, 0.10076)	0.00023 (0.00022, 0.00024)	-1.00748 (-1.07040, -0.79875)	0.83519 (0.82017, 0.88501)
2019	0.10647 (0.08459, 0.11271)	0.13009 (0.11703, 0.14025)	0.00048 (0.00037, 0.00061)	-0.40898 (-0.80236, -0.26985)	1.31870 (1.11288, 1.47186)
2020	0.04438 (0.04331,	0.06347 (0.05250,	0.00079 (0.00067,	1.26556 (0.76609,	1.01576 (0.93395,

	0.04796)	0.08597)	0.00102)	1.47357)	1.17164)
2021	0.09599 (0.09528, 0.09803)	0.12345 (0.11906, 0.12572)	0.00064 (0.00057, 0.00081)	-0.18543 (-0.19823, -0.17808)	1.76726 (1.52843, 1.88840)
2022	0.07748 (0.06106, 0.08062)	0.09248 (0.07447, 0.09885)	0.00077 (0.00033, 0.00081)	0.54658 (0.45090, 1.02975)	3.10805 (3.05674, 3.13918)
2023	0.07819 (0.06388, 0.09680)	0.10645 (0.09034, 0.11877)	0.00222 (0.00164, 0.00242)	0.23005 (0.20654, 0.29140)	3.08082 (3.02621, 3.11592)
2024	0.08165 (0.08165, 0.08165)	0.13048 (0.13048, 0.13048)	0.00104 (0.00104, 0.00104)	-0.11935 (-0.11935, -0.11935)	1.82299 (1.82299, 1.82299)

Table S3 Threshold Vaccine Price for Bivalent EV-A71/CV-A16 Vaccine under Different Strategies and Willingness-to-Pay Levels

strategy	coverage	VE	TVP1	TVP2	TVP3
Strategy 1	0.2	0.5	77.2(73.6,80.7)	85.3(81.7,88.8)	93.4(89.8,96.9)
Strategy 1	0.2	0.55	78.9(75.3,82.4)	87.5(83.9,91)	96.1(92.6,99.7)
Strategy 1	0.2	0.6	80.3(76.8,83.9)	89.4(85.8,92.9)	98.4(94.9,102)
Strategy 1	0.2	0.65	81.5(78,85.1)	91(87.4,94.5)	100.4(96.9,104)
Strategy 1	0.2	0.7	82.6(79,86.1)	92.3(88.8,95.9)	102.1(98.5,105.6)
Strategy 1	0.2	0.75	83.4(79.9,87)	93.5(89.9,97)	103.5(99.9,107)
Strategy 1	0.2	0.8	84.2(80.6,87.7)	94.4(90.9,98)	104.7(101.1,108.2)
Strategy 1	0.2	0.85	84.8(81.2,88.4)	95.2(91.7,98.8)	105.7(102.1,109.2)
Strategy 1	0.2	0.9	85.3(81.8,88.9)	95.9(92.4,99.5)	106.6(103,110.1)
Strategy 1	0.2	0.95	85.8(82.2,89.4)	96.6(93,100.1)	107.3(103.8,110.9)
Strategy 1	0.25	0.5	57.2(54.4,60.1)	64.6(61.8,67.5)	72(69.2,74.9)
Strategy 1	0.25	0.55	58.3(55.5,61.2)	66.1(63.2,68.9)	73.8(71,76.7)
Strategy 1	0.25	0.6	59.2(56.4,62.1)	67.2(64.4,70.1)	75.3(72.4,78.1)
Strategy 1	0.25	0.65	60(57.1,62.8)	68.2(65.3,71.1)	76.5(73.6,79.3)
Strategy 1	0.25	0.7	60.5(57.7,63.4)	69(66.1,71.8)	77.4(74.6,80.3)
Strategy 1	0.25	0.75	61(58.2,63.9)	69.6(66.8,72.5)	78.2(75.4,81.1)
Strategy 1	0.25	0.8	61.5(58.6,64.3)	70.2(67.3,73)	78.9(76,81.8)
Strategy 1	0.25	0.85	61.8(59,64.7)	70.6(67.8,73.5)	79.5(76.6,82.3)
Strategy 1	0.25	0.9	62.1(59.3,65)	71(68.2,73.9)	80(77.1,82.8)
Strategy 1	0.25	0.95	62.4(59.5,65.2)	71.4(68.5,74.2)	80.4(77.5,83.3)
Strategy 1	0.3	0.5	42.5(40.1,44.8)	49.1(46.8,51.5)	55.8(53.4,58.1)
Strategy 1	0.3	0.55	43.2(40.8,45.5)	50(47.7,52.4)	56.9(54.6,59.3)
Strategy 1	0.3	0.6	43.7(41.4,46.1)	50.8(48.4,53.1)	57.8(55.5,60.2)

Strategy 1	0.3	0.65	44.2(41.8,46.5)	51.4(49,53.7)	58.6(56.2,60.9)
Strategy 1	0.3	0.7	44.5(42.2,46.9)	51.8(49.5,54.2)	59.1(56.8,61.5)
Strategy 1	0.3	0.75	44.8(42.5,47.2)	52.2(49.9,54.6)	59.6(57.3,62)
Strategy 1	0.3	0.8	45.1(42.7,47.5)	52.6(50.2,55)	60.1(57.7,62.4)
Strategy 1	0.3	0.85	45.3(43,47.7)	52.9(50.5,55.2)	60.4(58.1,62.8)
Strategy 1	0.3	0.9	45.5(43.1,47.9)	53.1(50.8,55.5)	60.7(58.4,63.1)
Strategy 1	0.3	0.95	45.7(43.3,48.1)	53.4(51,55.7)	61(58.6,63.4)
Strategy 1	0.35	0.5	31.9(29.9,33.9)	37.9(35.9,39.9)	43.9(41.9,46)
Strategy 1	0.35	0.55	32.4(30.3,34.4)	38.5(36.5,40.6)	44.7(42.6,46.7)
Strategy 1	0.35	0.6	32.7(30.7,34.8)	39(37,41.1)	45.3(43.3,47.3)
Strategy 1	0.35	0.65	33(31,35.1)	39.4(37.4,41.5)	45.8(43.8,47.8)
Strategy 1	0.35	0.7	33.3(31.3,35.3)	39.7(37.7,41.8)	46.2(44.2,48.2)
Strategy 1	0.35	0.75	33.5(31.5,35.5)	40(38,42.1)	46.5(44.5,48.6)
Strategy 1	0.35	0.8	33.7(31.7,35.7)	40.3(38.2,42.3)	46.8(44.8,48.9)
Strategy 1	0.35	0.85	33.8(31.8,35.9)	40.5(38.4,42.5)	47.1(45,49.1)
Strategy 1	0.35	0.9	34(31.9,36)	40.6(38.6,42.7)	47.3(45.3,49.3)
Strategy 1	0.35	0.95	34.1(32.1,36.1)	40.8(38.8,42.8)	47.5(45.5,49.5)
Strategy 1	0.4	0.5	23.7(21.9,25.5)	29.1(27.3,30.9)	34.6(32.8,36.4)
Strategy 1	0.4	0.55	24(22.2,25.8)	29.6(27.8,31.4)	35.1(33.3,36.9)
Strategy 1	0.4	0.6	24.3(22.5,26.1)	29.9(28.1,31.7)	35.5(33.8,37.3)
Strategy 1	0.4	0.65	24.5(22.7,26.3)	30.2(28.4,32)	35.9(34.1,37.7)
Strategy 1	0.4	0.7	24.7(22.9,26.5)	30.4(28.7,32.2)	36.2(34.4,38)
Strategy 1	0.4	0.75	24.8(23.1,26.6)	30.6(28.9,32.4)	36.5(34.7,38.2)
Strategy 1	0.4	0.8	25(23.2,26.8)	30.8(29,32.6)	36.7(34.9,38.5)
Strategy 1	0.4	0.85	25.1(23.3,26.9)	31(29.2,32.8)	36.9(35.1,38.6)

Strategy 1	0.4	0.9	25.2(23.4,27)	31.1(29.3,32.9)	37(35.2,38.8)
Strategy 1	0.4	0.95	25.3(23.5,27.1)	31.2(29.4,33)	37.2(35.4,39)
Strategy 1	0.45	0.5	17.1(15.5,18.7)	22.1(20.5,23.7)	27(25.4,28.6)
Strategy 1	0.45	0.55	17.4(15.8,19)	22.4(20.8,24)	27.4(25.9,29)
Strategy 1	0.45	0.6	17.6(16,19.2)	22.7(21.1,24.3)	27.8(26.2,29.4)
Strategy 1	0.45	0.65	17.8(16.2,19.3)	22.9(21.3,24.5)	28(26.5,29.6)
Strategy 1	0.45	0.7	17.9(16.3,19.5)	23.1(21.5,24.7)	28.3(26.7,29.9)
Strategy 1	0.45	0.75	18(16.4,19.6)	23.2(21.6,24.8)	28.5(26.9,30.1)
Strategy 1	0.45	0.8	18.1(16.5,19.7)	23.4(21.8,25)	28.6(27,30.2)
Strategy 1	0.45	0.85	18.2(16.6,19.8)	23.5(21.9,25.1)	28.8(27.2,30.4)
Strategy 1	0.45	0.9	18.3(16.7,19.9)	23.6(22,25.2)	28.9(27.3,30.5)
Strategy 1	0.45	0.95	18.4(16.8,20)	23.7(22.1,25.3)	29(27.4,30.6)
Strategy 1	0.5	0.5	11.4(10,12.8)	15.9(14.5,17.3)	20.4(19,21.8)
Strategy 1	0.5	0.55	11.6(10.2,13)	16.1(14.7,17.6)	20.7(19.3,22.1)
Strategy 1	0.5	0.6	11.7(10.3,13.2)	16.3(14.9,17.8)	21(19.5,22.4)
Strategy 1	0.5	0.65	11.9(10.4,13.3)	16.5(15.1,17.9)	21.2(19.8,22.6)
Strategy 1	0.5	0.7	12(10.5,13.4)	16.7(15.2,18.1)	21.3(19.9,22.8)
Strategy 1	0.5	0.75	12.1(10.6,13.5)	16.8(15.4,18.2)	21.5(20.1,22.9)
Strategy 1	0.5	0.8	12.1(10.7,13.6)	16.9(15.5,18.3)	21.6(20.2,23.1)
Strategy 1	0.5	0.85	12.2(10.8,13.6)	17(15.6,18.4)	21.8(20.3,23.2)
Strategy 1	0.5	0.9	12.3(10.9,13.7)	17.1(15.6,18.5)	21.9(20.4,23.3)
Strategy 1	0.5	0.95	12.3(10.9,13.8)	17.1(15.7,18.6)	22(20.5,23.4)
Strategy 1	0.55	0.5	7(5.7,8.3)	11.2(9.9,12.5)	15.3(14,16.6)
Strategy 1	0.55	0.55	7.2(5.9,8.5)	11.4(10.1,12.7)	15.6(14.3,16.9)
Strategy 1	0.55	0.6	7.3(6,8.6)	11.6(10.3,12.8)	15.8(14.5,17.1)

Strategy 1	0.55	0.65	7.4(6.1,8.7)	11.7(10.4,13)	16(14.7,17.3)
Strategy 1	0.55	0.7	7.5(6.2,8.8)	11.8(10.5,13.1)	16.1(14.8,17.4)
Strategy 1	0.55	0.75	7.6(6.3,8.9)	11.9(10.6,13.2)	16.2(14.9,17.5)
Strategy 1	0.55	0.8	7.6(6.3,8.9)	12(10.7,13.3)	16.3(15.1,17.6)
Strategy 1	0.55	0.85	7.7(6.4,9)	12.1(10.8,13.4)	16.4(15.2,17.7)
Strategy 1	0.55	0.9	7.8(6.5,9.1)	12.1(10.8,13.4)	16.5(15.2,17.8)
Strategy 1	0.55	0.95	7.8(6.5,9.1)	12.2(10.9,13.5)	16.6(15.3,17.9)
Strategy 1	0.6	0.5	3.4(2.2,4.5)	7.2(6,8.4)	11.1(9.9,12.2)
Strategy 1	0.6	0.55	3.5(2.3,4.7)	7.4(6.2,8.6)	11.3(10.1,12.5)
Strategy 1	0.6	0.6	3.6(2.4,4.8)	7.5(6.3,8.7)	11.4(10.2,12.6)
Strategy 1	0.6	0.65	3.7(2.5,4.9)	7.6(6.4,8.8)	11.6(10.4,12.8)
Strategy 1	0.6	0.7	3.7(2.6,4.9)	7.7(6.5,8.9)	11.7(10.5,12.9)
Strategy 1	0.6	0.75	3.8(2.6,5)	7.8(6.6,9)	11.8(10.6,13)
Strategy 1	0.6	0.8	3.9(2.7,5.1)	7.9(6.7,9.1)	11.9(10.7,13.1)
Strategy 1	0.6	0.85	3.9(2.7,5.1)	8(6.8,9.1)	12(10.8,13.2)
Strategy 1	0.6	0.9	4(2.8,5.2)	8(6.8,9.2)	12.1(10.9,13.3)
Strategy 1	0.6	0.95	4(2.8,5.2)	8.1(6.9,9.3)	12.1(10.9,13.3)
Strategy 1	0.65	0.5	0.1(-1,1.2)	3.7(2.6,4.8)	7.2(6.2,8.3)
Strategy 1	0.65	0.55	0.2(-0.9,1.3)	3.8(2.7,4.9)	7.4(6.3,8.5)
Strategy 1	0.65	0.6	0.3(-0.8,1.4)	3.9(2.8,5)	7.6(6.5,8.7)
Strategy 1	0.65	0.65	0.4(-0.7,1.4)	4(2.9,5.1)	7.7(6.6,8.8)
Strategy 1	0.65	0.7	0.4(-0.7,1.5)	4.1(3,5.2)	7.8(6.7,8.9)
Strategy 1	0.65	0.75	0.5(-0.6,1.6)	4.2(3.1,5.3)	7.9(6.8,9)
Strategy 1	0.65	0.8	0.5(-0.6,1.6)	4.2(3.1,5.3)	8(6.9,9.1)
Strategy 1	0.65	0.85	0.6(-0.5,1.7)	4.3(3.2,5.4)	8(6.9,9.1)

Strategy 1	0.65	0.9	0.6(-0.5,1.7)	4.4(3.3,5.4)	8.1(7,9.2)
Strategy 1	0.65	0.95	0.6(-0.5,1.7)	4.4(3.3,5.5)	8.2(7.1,9.3)
Strategy 1	0.7	0.5	-2.6(-3.6,-1.6)	0.8(-0.3,1.8)	4.1(3.1,5.1)
Strategy 1	0.7	0.55	-2.5(-3.5,-1.5)	0.9(-0.1,1.9)	4.2(3.2,5.3)
Strategy 1	0.7	0.6	-2.4(-3.5,-1.4)	1(0,2)	4.4(3.4,5.4)
Strategy 1	0.7	0.65	-2.4(-3.4,-1.4)	1(0,2.1)	4.5(3.5,5.5)
Strategy 1	0.7	0.7	-2.3(-3.3,-1.3)	1.1(0.1,2.1)	4.6(3.6,5.6)
Strategy 1	0.7	0.75	-2.3(-3.3,-1.3)	1.2(0.2,2.2)	4.7(3.6,5.7)
Strategy 1	0.7	0.8	-2.2(-3.2,-1.2)	1.2(0.2,2.3)	4.7(3.7,5.7)
Strategy 1	0.7	0.85	-2.2(-3.2,-1.2)	1.3(0.3,2.3)	4.8(3.8,5.8)
Strategy 1	0.7	0.9	-2.2(-3.2,-1.1)	1.3(0.3,2.4)	4.8(3.8,5.9)
Strategy 1	0.7	0.95	-2.1(-3.1,-1.1)	1.4(0.4,2.4)	4.9(3.9,5.9)
Strategy 1	0.75	0.5	-5(-5.9,-4)	-1.8(-2.8,-0.8)	1.4(0.4,2.3)
Strategy 1	0.75	0.55	-4.9(-5.8,-3.9)	-1.7(-2.6,-0.7)	1.5(0.5,2.4)
Strategy 1	0.75	0.6	-4.8(-5.8,-3.9)	-1.6(-2.6,-0.7)	1.6(0.6,2.5)
Strategy 1	0.75	0.65	-4.8(-5.7,-3.8)	-1.5(-2.5,-0.6)	1.7(0.7,2.6)
Strategy 1	0.75	0.7	-4.7(-5.7,-3.8)	-1.5(-2.4,-0.5)	1.8(0.8,2.7)
Strategy 1	0.75	0.75	-4.7(-5.6,-3.7)	-1.4(-2.4,-0.5)	1.8(0.9,2.8)
Strategy 1	0.75	0.8	-4.6(-5.6,-3.7)	-1.4(-2.3,-0.4)	1.9(0.9,2.8)
Strategy 1	0.75	0.85	-4.6(-5.5,-3.6)	-1.3(-2.3,-0.4)	1.9(1,2.9)
Strategy 1	0.75	0.9	-4.6(-5.5,-3.6)	-1.3(-2.2,-0.3)	2(1,3)
Strategy 1	0.75	0.95	-4.5(-5.5,-3.6)	-1.2(-2.2,-0.3)	2(1.1,3)
Strategy 1	0.8	0.5	-7(-7.9,-6.1)	-4(-4.9,-3.2)	-1.1(-2,-0.2)
Strategy 1	0.8	0.55	-7(-7.8,-6.1)	-4(-4.8,-3.1)	-1(-1.9,-0.1)
Strategy 1	0.8	0.6	-6.9(-7.8,-6)	-3.9(-4.8,-3)	-0.9(-1.8,0)

Strategy 1	0.8	0.65	-6.8(-7.7,-6)	-3.8(-4.7,-2.9)	-0.8(-1.7,0.1)
Strategy 1	0.8	0.7	-6.8(-7.7,-5.9)	-3.8(-4.7,-2.9)	-0.7(-1.6,0.2)
Strategy 1	0.8	0.75	-6.8(-7.7,-5.9)	-3.7(-4.6,-2.8)	-0.7(-1.5,0.2)
Strategy 1	0.8	0.8	-6.7(-7.6,-5.8)	-3.7(-4.6,-2.8)	-0.6(-1.5,0.3)
Strategy 1	0.8	0.85	-6.7(-7.6,-5.8)	-3.6(-4.5,-2.7)	-0.5(-1.4,0.4)
Strategy 1	0.8	0.9	-6.7(-7.6,-5.8)	-3.6(-4.5,-2.7)	-0.5(-1.4,0.4)
Strategy 1	0.8	0.95	-6.7(-7.5,-5.8)	-3.6(-4.4,-2.7)	-0.5(-1.4,0.4)
Strategy 1	0.85	0.5	-9(-9.8,-8.2)	-6.2(-7,-5.4)	-3.4(-4.2,-2.6)
Strategy 1	0.85	0.55	-8.9(-9.8,-8.1)	-6.1(-7,-5.3)	-3.3(-4.1,-2.5)
Strategy 1	0.85	0.6	-8.9(-9.7,-8.1)	-6.1(-6.9,-5.2)	-3.2(-4.1,-2.4)
Strategy 1	0.85	0.65	-8.9(-9.7,-8)	-6(-6.8,-5.2)	-3.1(-4,-2.3)
Strategy 1	0.85	0.7	-8.8(-9.7,-8)	-6(-6.8,-5.1)	-3.1(-3.9,-2.2)
Strategy 1	0.85	0.75	-8.8(-9.6,-7.9)	-5.9(-6.7,-5.1)	-3(-3.9,-2.2)
Strategy 1	0.85	0.8	-8.8(-9.6,-7.9)	-5.9(-6.7,-5)	-3(-3.8,-2.1)
Strategy 1	0.85	0.85	-8.7(-9.6,-7.9)	-5.8(-6.7,-5)	-2.9(-3.8,-2.1)
Strategy 1	0.85	0.9	-8.7(-9.5,-7.9)	-5.8(-6.6,-5)	-2.9(-3.7,-2.1)
Strategy 1	0.85	0.95	-8.7(-9.5,-7.8)	-5.8(-6.6,-4.9)	-2.9(-3.7,-2)
Strategy 1	0.9	0.5	-10.6(-11.4,-9.8)	-8(-8.8,-7.2)	-5.3(-6.1,-4.5)
Strategy 1	0.9	0.55	-10.6(-11.4,-9.8)	-7.9(-8.7,-7.1)	-5.2(-6,-4.4)
Strategy 1	0.9	0.6	-10.5(-11.3,-9.7)	-7.8(-8.6,-7)	-5.1(-5.9,-4.3)
Strategy 1	0.9	0.65	-10.5(-11.3,-9.7)	-7.8(-8.6,-7)	-5.1(-5.9,-4.3)
Strategy 1	0.9	0.7	-10.5(-11.2,-9.7)	-7.7(-8.5,-6.9)	-5(-5.8,-4.2)
Strategy 1	0.9	0.75	-10.4(-11.2,-9.6)	-7.7(-8.5,-6.9)	-5(-5.8,-4.2)
Strategy 1	0.9	0.8	-10.4(-11.2,-9.6)	-7.7(-8.5,-6.9)	-4.9(-5.7,-4.1)
Strategy 1	0.9	0.85	-10.4(-11.2,-9.6)	-7.6(-8.4,-6.8)	-4.9(-5.7,-4.1)

Strategy 1	0.9	0.9	-10.3(-11.1,-9.6)	-7.6(-8.4,-6.8)	-4.8(-5.6,-4.1)
Strategy 1	0.9	0.95	-10.3(-11.1,-9.5)	-7.6(-8.4,-6.8)	-4.8(-5.6,-4)
Strategy 1	0.95	0.5	-12.1(-12.8,-11.3)	-9.6(-10.3,-8.8)	-7(-7.8,-6.3)
Strategy 1	0.95	0.55	-12(-12.8,-11.3)	-9.5(-10.2,-8.7)	-6.9(-7.7,-6.2)
Strategy 1	0.95	0.6	-12(-12.8,-11.2)	-9.4(-10.2,-8.7)	-6.9(-7.6,-6.1)
Strategy 1	0.95	0.65	-12(-12.7,-11.2)	-9.4(-10.1,-8.6)	-6.8(-7.6,-6.1)
Strategy 1	0.95	0.7	-11.9(-12.7,-11.2)	-9.3(-10.1,-8.6)	-6.8(-7.5,-6)
Strategy 1	0.95	0.75	-11.9(-12.7,-11.2)	-9.3(-10.1,-8.6)	-6.7(-7.5,-6)
Strategy 1	0.95	0.8	-11.9(-12.6,-11.1)	-9.3(-10,-8.5)	-6.7(-7.4,-5.9)
Strategy 1	0.95	0.85	-11.9(-12.6,-11.1)	-9.2(-10,-8.5)	-6.6(-7.4,-5.9)
Strategy 1	0.95	0.9	-11.8(-12.6,-11.1)	-9.2(-10,-8.5)	-6.6(-7.4,-5.8)
Strategy 1	0.95	0.95	-11.8(-12.6,-11.1)	-9.2(-9.9,-8.4)	-6.6(-7.3,-5.8)
Strategy 2	0.2	0.5	231(221.5,240.6)	239.8(230.3,249.3)	248.6(239,258.1)
Strategy 2	0.2	0.55	234.1(224.6,243.7)	243.8(234.3,253.4)	253.5(244,263.1)
Strategy 2	0.2	0.6	237.1(227.6,246.7)	247.8(238.3,257.4)	258.5(248.9,268)
Strategy 2	0.2	0.65	240.2(230.6,249.7)	251.8(242.2,261.3)	263.3(253.8,272.9)
Strategy 2	0.2	0.7	243.1(233.6,252.7)	255.6(246.1,265.2)	268.1(258.6,277.7)
Strategy 2	0.2	0.75	246.1(236.5,255.6)	259.5(249.9,269)	272.9(263.3,282.4)
Strategy 2	0.2	0.8	248.9(239.4,258.5)	263.2(253.7,272.8)	277.5(268,287.1)
Strategy 2	0.2	0.85	251.7(242.2,261.3)	266.9(257.3,276.4)	282(272.5,291.6)
Strategy 2	0.2	0.9	254.5(244.9,264)	270.4(260.9,280)	286.4(276.9,296)
Strategy 2	0.2	0.95	257.1(247.6,266.7)	273.9(264.3,283.4)	290.7(281.1,300.2)
Strategy 2	0.25	0.5	181.8(174.2,189.4)	190.7(183.1,198.3)	199.5(191.9,207.1)
Strategy 2	0.25	0.55	184.8(177.2,192.4)	194.5(187,202.2)	204.3(196.7,211.9)
Strategy 2	0.25	0.6	187.7(180.1,195.3)	198.4(190.8,206)	209(201.4,216.6)

Strategy 2	0.25	0.65	190.5(183,198.2)	202.1(194.5,209.7)	213.6(206,221.2)
Strategy 2	0.25	0.7	193.3(185.7,200.9)	205.7(198.1,213.3)	218(210.4,225.6)
Strategy 2	0.25	0.75	196(188.4,203.6)	209.1(201.6,216.7)	222.3(214.7,229.9)
Strategy 2	0.25	0.8	198.5(190.9,206.1)	212.5(204.9,220.1)	226.4(218.8,234)
Strategy 2	0.25	0.85	200.9(193.4,208.5)	215.6(208.1,223.2)	230.3(222.8,237.9)
Strategy 2	0.25	0.9	203.2(195.7,210.8)	218.7(211.1,226.3)	234.1(226.5,241.7)
Strategy 2	0.25	0.95	205.4(197.8,213)	221.5(213.9,229.1)	237.6(230,245.2)
Strategy 2	0.3	0.5	149.2(142.9,155.5)	158(151.7,164.3)	166.8(160.5,173.1)
Strategy 2	0.3	0.55	152(145.7,158.3)	161.7(155.4,168)	171.4(165.1,177.7)
Strategy 2	0.3	0.6	154.7(148.4,161)	165.2(158.9,171.5)	175.8(169.5,182.1)
Strategy 2	0.3	0.65	157.3(151,163.6)	168.6(162.3,174.9)	179.9(173.6,186.2)
Strategy 2	0.3	0.7	159.7(153.4,166.1)	171.8(165.5,178.1)	183.9(177.6,190.2)
Strategy 2	0.3	0.75	162.1(155.8,168.4)	174.8(168.5,181.2)	187.6(181.3,193.9)
Strategy 2	0.3	0.8	164.2(157.9,170.5)	177.7(171.4,184)	191.1(184.8,197.4)
Strategy 2	0.3	0.85	166.2(159.9,172.5)	180.3(174,186.6)	194.3(188.1,200.7)
Strategy 2	0.3	0.9	168.1(161.8,174.4)	182.7(176.4,189)	197.3(191,203.7)
Strategy 2	0.3	0.95	169.8(163.5,176.1)	184.9(178.6,191.2)	200.1(193.8,206.4)
Strategy 2	0.35	0.5	128(122.6,133.5)	136.8(131.3,142.3)	145.6(140.1,151.1)
Strategy 2	0.35	0.55	130.7(125.2,136.2)	140.3(134.8,145.8)	149.8(144.4,155.3)
Strategy 2	0.35	0.6	133.2(127.7,138.7)	143.5(138.1,149)	153.9(148.4,159.4)
Strategy 2	0.35	0.65	135.5(130,141)	146.6(141.1,152.1)	157.6(152.2,163.1)
Strategy 2	0.35	0.7	137.7(132.2,143.2)	149.4(143.9,154.9)	161.1(155.6,166.6)
Strategy 2	0.35	0.75	139.6(134.2,145.1)	152(146.5,157.5)	164.3(158.8,169.8)
Strategy 2	0.35	0.8	141.4(136,146.9)	154.3(148.9,159.8)	167.2(161.7,172.7)
Strategy 2	0.35	0.85	143.1(137.6,148.6)	156.5(151,161.9)	169.8(164.4,175.3)

Strategy 2	0.35	0.9	144.6(139.1,150)	158.4(152.9,163.9)	172.2(166.8,177.7)
Strategy 2	0.35	0.95	145.9(140.4,151.4)	160.1(154.6,165.6)	174.4(168.9,179.8)
Strategy 2	0.4	0.5	111.6(106.8,116.4)	120.2(115.4,125.1)	128.9(124.1,133.8)
Strategy 2	0.4	0.55	114(109.2,118.9)	123.4(118.6,128.3)	132.8(128,137.7)
Strategy 2	0.4	0.6	116.3(111.4,121.1)	126.3(121.5,131.2)	136.4(131.6,141.3)
Strategy 2	0.4	0.65	118.3(113.5,123.2)	129(124.2,133.9)	139.7(134.9,144.6)
Strategy 2	0.4	0.7	120.1(115.3,125)	131.4(126.6,136.3)	142.7(137.9,147.6)
Strategy 2	0.4	0.75	121.8(117,126.6)	133.6(128.7,138.4)	145.4(140.5,150.2)
Strategy 2	0.4	0.8	123.3(118.4,128.1)	135.5(130.7,140.3)	147.7(142.9,152.6)
Strategy 2	0.4	0.85	124.6(119.7,129.4)	137.2(132.4,142.1)	149.8(145,154.7)
Strategy 2	0.4	0.9	125.7(120.9,130.6)	138.7(133.9,143.6)	151.7(146.9,156.5)
Strategy 2	0.4	0.95	126.7(121.9,131.6)	140(135.2,144.9)	153.3(148.5,158.2)
Strategy 2	0.45	0.5	96.9(92.6,101.2)	105.4(101.1,109.7)	113.9(109.6,118.2)
Strategy 2	0.45	0.55	99.1(94.8,103.4)	108.2(104,112.5)	117.4(113.1,121.7)
Strategy 2	0.45	0.6	101(96.7,105.3)	110.8(106.5,115.1)	120.5(116.2,124.8)
Strategy 2	0.45	0.65	102.7(98.5,107)	113(108.7,117.3)	123.3(119,127.6)
Strategy 2	0.45	0.7	104.3(100,108.5)	115(110.7,119.3)	125.7(121.5,130)
Strategy 2	0.45	0.75	105.6(101.3,109.9)	116.7(112.4,121)	127.9(123.6,132.2)
Strategy 2	0.45	0.8	106.7(102.4,111)	118.2(114,122.5)	129.7(125.4,134)
Strategy 2	0.45	0.85	107.7(103.4,112)	119.5(115.2,123.8)	131.3(127.1,135.6)
Strategy 2	0.45	0.9	108.6(104.3,112.9)	120.7(116.4,125)	132.7(128.5,137)
Strategy 2	0.45	0.95	109.3(105.1,113.6)	121.6(117.4,125.9)	133.9(129.7,138.2)
Strategy 2	0.5	0.5	85(81.2,88.9)	93.3(89.5,97.1)	101.6(97.8,105.4)
Strategy 2	0.5	0.55	86.9(83.1,90.8)	95.8(91.9,99.6)	104.6(100.8,108.5)
Strategy 2	0.5	0.6	88.6(84.7,92.4)	97.9(94.1,101.8)	107.3(103.5,111.2)

Strategy 2	0.5	0.65	90(86.2,93.8)	99.8(96,103.7)	109.6(105.8,113.5)
Strategy 2	0.5	0.7	91.2(87.4,95.1)	101.4(97.6,105.2)	111.6(107.8,115.4)
Strategy 2	0.5	0.75	92.3(88.4,96.1)	102.8(98.9,106.6)	113.3(109.4,117.1)
Strategy 2	0.5	0.8	93.2(89.3,97)	103.9(100.1,107.8)	114.7(110.9,118.6)
Strategy 2	0.5	0.85	93.9(90.1,97.8)	104.9(101.1,108.8)	116(112.1,119.8)
Strategy 2	0.5	0.9	94.6(90.7,98.4)	105.8(102,109.6)	117(113.2,120.9)
Strategy 2	0.5	0.95	95.1(91.3,99)	106.5(102.7,110.4)	117.9(114.1,121.8)
Strategy 2	0.55	0.5	75.1(71.6,78.6)	83.2(79.7,86.6)	91.2(87.7,94.7)
Strategy 2	0.55	0.55	76.7(73.3,80.2)	85.3(81.8,88.8)	93.8(90.4,97.3)
Strategy 2	0.55	0.6	78.1(74.7,81.6)	87.1(83.6,90.6)	96.1(92.6,99.6)
Strategy 2	0.55	0.65	79.3(75.8,82.8)	88.6(85.2,92.1)	98(94.5,101.4)
Strategy 2	0.55	0.7	80.3(76.8,83.8)	89.9(86.4,93.4)	99.6(96.1,103)
Strategy 2	0.55	0.75	81.1(77.6,84.6)	91(87.5,94.5)	100.9(97.4,104.4)
Strategy 2	0.55	0.8	81.8(78.3,85.3)	91.9(88.4,95.4)	102(98.5,105.5)
Strategy 2	0.55	0.85	82.4(78.9,85.9)	92.7(89.2,96.2)	103(99.5,106.5)
Strategy 2	0.55	0.9	82.9(79.4,86.4)	93.3(89.9,96.8)	103.8(100.3,107.3)
Strategy 2	0.55	0.95	83.3(79.9,86.8)	93.9(90.4,97.4)	104.5(101,108)
Strategy 2	0.6	0.5	66.7(63.5,69.9)	74.5(71.3,77.7)	82.3(79.1,85.4)
Strategy 2	0.6	0.55	68.1(64.9,71.3)	76.3(73.1,79.5)	84.5(81.3,87.7)
Strategy 2	0.6	0.6	69.2(66.1,72.4)	77.8(74.6,81)	86.4(83.2,89.6)
Strategy 2	0.6	0.65	70.2(67,73.4)	79(75.9,82.2)	87.9(84.7,91.1)
Strategy 2	0.6	0.7	71(67.8,74.2)	80.1(76.9,83.3)	89.2(86,92.4)
Strategy 2	0.6	0.75	71.6(68.5,74.8)	80.9(77.8,84.1)	90.2(87.1,93.4)
Strategy 2	0.6	0.8	72.2(69,75.4)	81.7(78.5,84.9)	91.1(88,94.3)
Strategy 2	0.6	0.85	72.7(69.5,75.8)	82.3(79.1,85.5)	91.9(88.7,95.1)

Strategy 2	0.6	0.9	73.1(69.9,76.3)	82.8(79.6,86)	92.6(89.4,95.7)
Strategy 2	0.6	0.95	73.4(70.2,76.6)	83.3(80.1,86.4)	93.1(89.9,96.3)
Strategy 2	0.65	0.5	60.2(57.2,63.1)	67.7(64.7,70.7)	75.2(72.3,78.2)
Strategy 2	0.65	0.55	61.4(58.4,64.3)	69.3(66.3,72.2)	77.2(74.2,80.1)
Strategy 2	0.65	0.6	62.3(59.4,65.3)	70.5(67.6,73.5)	78.7(75.8,81.7)
Strategy 2	0.65	0.65	63.1(60.2,66.1)	71.6(68.6,74.6)	80(77.1,83)
Strategy 2	0.65	0.7	63.8(60.8,66.8)	72.4(69.5,75.4)	81.1(78.1,84.1)
Strategy 2	0.65	0.75	64.3(61.4,67.3)	73.2(70.2,76.1)	82(79,84.9)
Strategy 2	0.65	0.8	64.8(61.8,67.8)	73.8(70.8,76.7)	82.7(79.8,85.7)
Strategy 2	0.65	0.85	65.2(62.2,68.2)	74.3(71.3,77.2)	83.3(80.4,86.3)
Strategy 2	0.65	0.9	65.5(62.6,68.5)	74.7(71.7,77.7)	83.9(80.9,86.8)
Strategy 2	0.65	0.95	65.8(62.9,68.8)	75.1(72.1,78)	84.3(81.4,87.3)
Strategy 2	0.7	0.5	53.7(51,56.5)	61(58.2,63.7)	68.2(65.5,71)
Strategy 2	0.7	0.55	54.7(52,57.5)	62.3(59.6,65)	69.8(67.1,72.6)
Strategy 2	0.7	0.6	55.5(52.8,58.3)	63.4(60.6,66.1)	71.2(68.4,73.9)
Strategy 2	0.7	0.65	56.2(53.5,58.9)	64.2(61.5,67)	72.2(69.5,75)
Strategy 2	0.7	0.7	56.7(54,59.5)	64.9(62.2,67.7)	73.1(70.4,75.8)
Strategy 2	0.7	0.75	57.2(54.5,59.9)	65.5(62.8,68.2)	73.8(71.1,76.6)
Strategy 2	0.7	0.8	57.6(54.8,60.3)	66(63.2,68.7)	74.4(71.7,77.2)
Strategy 2	0.7	0.85	57.9(55.1,60.6)	66.4(63.7,69.2)	74.9(72.2,77.7)
Strategy 2	0.7	0.9	58.1(55.4,60.9)	66.8(64,69.5)	75.4(72.6,78.1)
Strategy 2	0.7	0.95	58.4(55.6,61.1)	67.1(64.3,69.8)	75.8(73,78.5)
Strategy 2	0.75	0.5	48(45.5,50.6)	55(52.5,57.6)	62(59.4,64.5)
Strategy 2	0.75	0.55	48.9(46.3,51.4)	56.1(53.6,58.7)	63.4(60.8,65.9)
Strategy 2	0.75	0.6	49.6(47,52.1)	57(54.5,59.6)	64.4(61.9,67)

Strategy 2	0.75	0.65	50.1(47.6,52.7)	57.7(55.2,60.3)	65.3(62.8,67.9)
Strategy 2	0.75	0.7	50.6(48,53.1)	58.3(55.8,60.9)	66(63.5,68.6)
Strategy 2	0.75	0.75	50.9(48.4,53.5)	58.8(56.2,61.3)	66.6(64.1,69.2)
Strategy 2	0.75	0.8	51.2(48.7,53.8)	59.2(56.6,61.8)	67.2(64.6,69.7)
Strategy 2	0.75	0.85	51.5(49,54.1)	59.5(57,62.1)	67.6(65,70.1)
Strategy 2	0.75	0.9	51.7(49.2,54.3)	59.8(57.3,62.4)	68(65.4,70.5)
Strategy 2	0.75	0.95	51.9(49.4,54.5)	60.1(57.6,62.7)	68.3(65.7,70.8)
Strategy 2	0.8	0.5	43(40.6,45.4)	49.7(47.3,52.1)	56.4(54,58.8)
Strategy 2	0.8	0.55	43.7(41.3,46.1)	50.6(48.2,53)	57.5(55.1,59.9)
Strategy 2	0.8	0.6	44.3(41.9,46.7)	51.4(49,53.8)	58.5(56.1,60.8)
Strategy 2	0.8	0.65	44.7(42.4,47.1)	52(49.6,54.4)	59.2(56.8,61.6)
Strategy 2	0.8	0.7	45.1(42.7,47.5)	52.5(50.1,54.8)	59.8(57.4,62.2)
Strategy 2	0.8	0.75	45.4(43,47.8)	52.9(50.5,55.3)	60.3(57.9,62.7)
Strategy 2	0.8	0.8	45.7(43.3,48.1)	53.2(50.8,55.6)	60.7(58.4,63.1)
Strategy 2	0.8	0.85	45.9(43.5,48.3)	53.5(51.1,55.9)	61.1(58.7,63.5)
Strategy 2	0.8	0.9	46.1(43.7,48.5)	53.8(51.4,56.1)	61.4(59,63.8)
Strategy 2	0.8	0.95	46.3(43.9,48.7)	54(51.6,56.4)	61.7(59.3,64.1)
Strategy 2	0.85	0.5	38.5(36.2,40.7)	44.9(42.6,47.1)	51.3(49.1,53.6)
Strategy 2	0.85	0.55	39.1(36.8,41.3)	45.7(43.5,47.9)	52.3(50.1,54.6)
Strategy 2	0.85	0.6	39.6(37.3,41.8)	46.3(44.1,48.6)	53.1(50.9,55.3)
Strategy 2	0.85	0.65	39.9(37.7,42.2)	46.8(44.6,49.1)	53.7(51.5,56)
Strategy 2	0.85	0.7	40.3(38,42.5)	47.2(45,49.5)	54.2(52,56.5)
Strategy 2	0.85	0.75	40.5(38.3,42.8)	47.6(45.4,49.8)	54.7(52.4,56.9)
Strategy 2	0.85	0.8	40.8(38.5,43)	47.9(45.7,50.1)	55(52.8,57.3)
Strategy 2	0.85	0.85	41(38.7,43.2)	48.1(45.9,50.4)	55.4(53.1,57.6)

Strategy 2	0.85	0.9	41.1(38.9,43.4)	48.4(46.1,50.6)	55.6(53.4,57.9)
Strategy 2	0.85	0.95	41.3(39,43.5)	48.6(46.3,50.8)	55.9(53.6,58.1)
Strategy 2	0.9	0.5	34.8(32.7,36.9)	41(38.9,43.1)	47.2(45.1,49.3)
Strategy 2	0.9	0.55	35.4(33.2,37.5)	41.7(39.6,43.8)	48.1(46,50.2)
Strategy 2	0.9	0.6	35.8(33.6,37.9)	42.3(40.1,44.4)	48.8(46.6,50.9)
Strategy 2	0.9	0.65	36.1(34,38.2)	42.7(40.6,44.8)	49.3(47.2,51.4)
Strategy 2	0.9	0.7	36.4(34.3,38.5)	43.1(41,45.2)	49.8(47.6,51.9)
Strategy 2	0.9	0.75	36.6(34.5,38.8)	43.4(41.3,45.5)	50.1(48,52.3)
Strategy 2	0.9	0.8	36.8(34.7,39)	43.6(41.5,45.8)	50.5(48.3,52.6)
Strategy 2	0.9	0.85	37(34.9,39.1)	43.9(41.7,46)	50.7(48.6,52.9)
Strategy 2	0.9	0.9	37.1(35,39.3)	44.1(41.9,46.2)	51(48.9,53.1)
Strategy 2	0.9	0.95	37.3(35.2,39.4)	44.2(42.1,46.4)	51.2(49.1,53.3)
Strategy 2	0.95	0.5	31.1(29.1,33.1)	37(35,39.1)	43(41,45)
Strategy 2	0.95	0.55	31.6(29.6,33.6)	37.7(35.6,39.7)	43.8(41.7,45.8)
Strategy 2	0.95	0.6	31.9(29.9,33.9)	38.1(36.1,40.1)	44.3(42.3,46.4)
Strategy 2	0.95	0.65	32.2(30.2,34.2)	38.5(36.5,40.5)	44.8(42.8,46.8)
Strategy 2	0.95	0.7	32.5(30.4,34.5)	38.8(36.8,40.9)	45.2(43.2,47.2)
Strategy 2	0.95	0.75	32.7(30.7,34.7)	39.1(37.1,41.1)	45.5(43.5,47.6)
Strategy 2	0.95	0.8	32.8(30.8,34.9)	39.3(37.3,41.4)	45.8(43.8,47.9)
Strategy 2	0.95	0.85	33(31,35)	39.5(37.5,41.5)	46.1(44.1,48.1)
Strategy 2	0.95	0.9	33.1(31.1,35.1)	39.7(37.7,41.7)	46.3(44.3,48.3)
Strategy 2	0.95	0.95	33.2(31.2,35.3)	39.9(37.9,41.9)	46.5(44.5,48.5)
Strategy 3	-	0.5	-13(-13.7,-12.3)	-10.5(-11.3,-9.8)	-8.1(-8.8,-7.4)
Strategy 3	-	0.55	-12.9(-13.7,-12.2)	-10.5(-11.2,-9.8)	-8(-8.7,-7.3)
Strategy 3	-	0.6	-12.9(-13.6,-12.2)	-10.4(-11.2,-9.7)	-7.9(-8.7,-7.2)

Strategy 3	-	0.65	-12.9(-13.6,-12.1)	-10.4(-11.1,-9.7)	-7.9(-8.6,-7.2)
Strategy 3	-	0.7	-12.8(-13.6,-12.1)	-10.3(-11.1,-9.6)	-7.8(-8.6,-7.1)
Strategy 3	-	0.75	-12.8(-13.5,-12.1)	-10.3(-11,-9.6)	-7.8(-8.5,-7.1)
Strategy 3	-	0.8	-12.8(-13.5,-12.1)	-10.3(-11,-9.5)	-7.8(-8.5,-7)
Strategy 3	-	0.85	-12.8(-13.5,-12)	-10.2(-11,-9.5)	-7.7(-8.4,-7)
Strategy 3	-	0.9	-12.7(-13.5,-12)	-10.2(-10.9,-9.5)	-7.7(-8.4,-7)
Strategy 3	-	0.95	-12.7(-13.4,-12)	-10.2(-10.9,-9.5)	-7.7(-8.4,-6.9)
Strategy 4	-	0.5	-16.1(-16.7,-15.4)	-13.9(-14.5,-13.2)	-11.7(-12.3,-11)
Strategy 4	-	0.55	-16(-16.7,-15.4)	-13.8(-14.4,-13.2)	-11.6(-12.2,-11)
Strategy 4	-	0.6	-16(-16.6,-15.4)	-13.8(-14.4,-13.1)	-11.6(-12.2,-10.9)
Strategy 4	-	0.65	-16(-16.6,-15.3)	-13.8(-14.4,-13.1)	-11.5(-12.2,-10.9)
Strategy 4	-	0.7	-16(-16.6,-15.3)	-13.7(-14.4,-13.1)	-11.5(-12.1,-10.9)
Strategy 4	-	0.75	-15.9(-16.6,-15.3)	-13.7(-14.3,-13.1)	-11.5(-12.1,-10.8)
Strategy 4	-	0.8	-15.9(-16.6,-15.3)	-13.7(-14.3,-13)	-11.4(-12.1,-10.8)
Strategy 4	-	0.85	-15.9(-16.6,-15.3)	-13.7(-14.3,-13)	-11.4(-12.1,-10.8)
Strategy 4	-	0.9	-15.9(-16.5,-15.3)	-13.7(-14.3,-13)	-11.4(-12,-10.8)
Strategy 4	-	0.95	-15.9(-16.5,-15.3)	-13.6(-14.3,-13)	-11.4(-12,-10.7)
Strategy 5	-	0.5	-19.4(-19.9,-18.8)	-17.5(-18,-16.9)	-15.6(-16.1,-15)
Strategy 5	-	0.55	-19.4(-19.9,-18.8)	-17.4(-18,-16.9)	-15.5(-16.1,-15)
Strategy 5	-	0.6	-19.4(-19.9,-18.8)	-17.4(-18,-16.9)	-15.5(-16,-15)
Strategy 5	-	0.65	-19.4(-19.9,-18.8)	-17.4(-18,-16.9)	-15.5(-16,-14.9)
Strategy 5	-	0.7	-19.3(-19.9,-18.8)	-17.4(-17.9,-16.9)	-15.5(-16,-14.9)
Strategy 5	-	0.75	-19.3(-19.9,-18.8)	-17.4(-17.9,-16.9)	-15.5(-16,-14.9)
Strategy 5	-	0.8	-19.3(-19.9,-18.8)	-17.4(-17.9,-16.8)	-15.4(-16,-14.9)
Strategy 5	-	0.85	-19.3(-19.9,-18.8)	-17.4(-17.9,-16.8)	-15.4(-16,-14.9)

Strategy 5	-	0.9	-19.3(-19.9,-18.8)	-17.4(-17.9,-16.8)	-15.4(-16,-14.9)
Strategy 5	-	0.95	-19.3(-19.9,-18.8)	-17.4(-17.9,-16.8)	-15.4(-16,-14.9)
Strategy 6	-	0.5	-17.1(-17.7,-16.5)	-15.0(-15.6,-14.4)	-12.8(-13.4,-12.2)
Strategy 6	-	0.55	-17.1(-17.7,-16.5)	-14.9(-15.5,-14.3)	-12.8(-13.4,-12.2)
Strategy 6	-	0.6	-17.0(-17.7,-16.4)	-14.9(-15.5,-14.3)	-12.8(-13.4,-12.2)
Strategy 6	-	0.65	-17.0(-17.6,-16.4)	-14.9(-15.5,-14.3)	-12.7(-13.3,-12.1)
Strategy 6	-	0.7	-17.0(-17.6,-16.4)	-14.9(-15.5,-14.3)	-12.7(-13.3,-12.1)
Strategy 6	-	0.75	-17.0(-17.6,-16.4)	-14.8(-15.5,-14.2)	-12.7(-13.3,-12.1)
Strategy 6	-	0.8	-17.0(-17.6,-16.4)	-14.8(-15.4,-14.2)	-12.7(-13.3,-12.1)
Strategy 6	-	0.85	-17.0(-17.6,-16.4)	-14.8(-15.4,-14.2)	-12.7(-13.3,-12.1)
Strategy 6	-	0.9	-17.0(-17.6,-16.4)	-14.8(-15.4,-14.2)	-12.6(-13.3,-12.0)
Strategy 6	-	0.95	-17.0(-17.6,-16.4)	-14.8(-15.4,-14.2)	-12.6(-13.2,-12.0)
Strategy 7	-	0.5	-15.8(-16.4,-15.1)	-13.6(-14.2,-12.9)	-11.3(-12.0,-10.7)
Strategy 7	-	0.55	-15.7(-16.4,-15.1)	-13.5(-14.1,-12.8)	-11.3(-11.9,-10.6)
Strategy 7	-	0.6	-15.7(-16.3,-15.0)	-13.5(-14.1,-12.8)	-11.2(-11.9,-10.6)
Strategy 7	-	0.65	-15.7(-16.3,-15.0)	-13.4(-14.1,-12.8)	-11.2(-11.8,-10.5)
Strategy 7	-	0.7	-15.6(-16.3,-15.0)	-13.4(-14.0,-12.7)	-11.1(-11.8,-10.5)
Strategy 7	-	0.75	-15.6(-16.3,-15.0)	-13.4(-14.0,-12.7)	-11.1(-11.8,-10.5)
Strategy 7	-	0.8	-15.6(-16.2,-14.9)	-13.3(-14.0,-12.7)	-11.1(-11.7,-10.4)
Strategy 7	-	0.85	-15.6(-16.2,-14.9)	-13.3(-14.0,-12.7)	-11.0(-11.7,-10.4)
Strategy 7	-	0.9	-15.6(-16.2,-14.9)	-13.3(-13.9,-12.6)	-11.0(-11.7,-10.4)
Strategy 7	-	0.95	-15.5(-16.2,-14.9)	-13.3(-13.9,-12.6)	-11.0(-11.6,-10.3)
